# The reach, metric distortion, geodesic convexity and the variation of tangent spaces

**DOI:** 10.1007/s41468-019-00029-8

**Published:** 2019-07-24

**Authors:** Jean-Daniel Boissonnat, André Lieutier, Mathijs Wintraecken

**Affiliations:** 1 Inria Sophia Antipolis Méditerranée, Université Côte d’Azur, 2004 Route des Lucioles, 06902 Valbonne, France; 2 Institute of Science and Technology (IST Austria), Am Campus 1, 3400 Klosterneuburg, Austria; 3 Dassault Systèmes, 53 Avenue de l’Europe, 13090 Aix-en-Provence, France

**Keywords:** Reach, Metric distortion, Manifolds, Convexity, 53C22 Geodesics, 53B25 Local submanifolds

## Abstract

In this paper we discuss three results. The first two concern general sets of positive reach: we first characterize the reach of a closed set by means of a bound on the metric distortion between the distance measured in the ambient Euclidean space and the shortest path distance measured in the set. Secondly, we prove that the intersection of a ball with radius less than the reach with the set is geodesically convex, meaning that the shortest path between any two points in the intersection lies itself in the intersection. For our third result we focus on manifolds with positive reach and give a bound on the angle between tangent spaces at two different points in terms of the reach and the distance between the two points.

## Introduction

Metric distortion quantifies the maximum ratio between geodesic and Euclidean distances for pairs of points in a set $\mathcal {S}$. The reach of $\mathcal {S}$, defined by Federer (1959), is the infimum of distances between points in $\mathcal {S}$ and points in its medial axis, the points in ambient space for which there does not exist a unique closest point in $\mathcal {S}$. Both reach and metric distortion are central concepts in manifold (re-)construction and have been used to characterize the size of topological features. Amenta and Bern (1999) introduced a local version of the reach in order to give conditions for homeomorphic surface reconstruction and this criterion has been used in many works aiming at topologically faithful reconstruction. See the seminal paper of Niyogi et al. (2008) and Dey (2006) for more context and references. A direct relation between the reach and the size of topological features is simply illustrated by the fact that the intersection of a set with reach $r>0$ with a ball of radius less than *r* has reach at least *r* and is contractible (Attali and Lieutier 2015). In a certain way, metric distortion also characterizes the size of topological features. This is illustrated by the fact that a compact subset of $\mathbb {R}^n$ with metric distortion less than $\pi /2$ is simply connected [section 1.14 in Gromov et al. (2007), see also appendix A by P. Pansu where sets with a given metric distortion are called *quasi convex sets*].

In the first part of this paper, we provide tight bounds on metric distortion for sets of positive reach and, in a second part, we consider submanifolds of $\mathbb {R}^d$ and bound the angle between tangent spaces at different points. Whenever we mention manifolds we shall tacitly assume that it is embedded in Euclidean space. Previous versions of the metric distortion result, restricted to the manifold setting can be found in Niyogi et al. (2008). A significant amount of attention has gone to tangent space variation, see Belkin et al. (2009), Boissonnat et al. (2013), Boissonnat and Ghosh (2010), Cheng et al. (2005), Dey (2006), Dey et al. (2008) and Niyogi et al. (2008) to name but a few.

Our paper improves on these bounds, extends the results beyond the case of smooth manifolds and offers new insights and results. These results have immediate algorithmic consequences by, on one hand, improving the sampling conditions under which known reconstruction algorithms are valid and, on the other hand, allowing us to extend the algorithms to the class of manifolds of positive reach, which is much larger than the usually considered class of $C^2$ manifolds. Indeed, the metric distortion and tangent variation bounds for $C^{1,1}$ manifolds presented in this paper in fact suffice to extend the triangulation result of $C^2$ manifolds embedded in Euclidean space given in Boissonnat et al. (2018) to arbitrary manifolds with positive reach, albeit with slightly worse constants. The results of the papers on manifold reconstruction cited above generalize likewise to general manifolds of positive reach. The constants that appear in the conditions that guarantee correctness of the papers above can also be improved in the $C^2$ case using the results presented here.

*Overview of results* For metric distortion, we extend and tighten the previously known results so much that our metric distortion result can be regarded as a completely new characterization of sets of positive reach. In particular, the standard manifold and smoothness assumptions are no longer necessary. Based on our new characterization of the reach by metric distortion, we can prove that the intersection of a set of positive reach with a ball with radius less than the reach is geodesically convex. This result is a far reaching extension of a result of Boissonnat and Oudot (2003) that has attracted significant attention, stating that, for smooth surfaces, the intersection is a pseudo-ball.

To study tangent variation along manifolds, we will consider two different settings, namely the $C^2$ setting, for which the bounds are tight, and the $C^{1,1}$ setting, where we achieve slightly weaker bounds.

The exposition for $C^2$ manifolds is based on differential geometry and is a consequence of combining the work of Niyogi et al. (2008), and the two dimensional analysis of Attali et al. (2007) with some observations concerning the reach. We would like to stress that some effort went into simplifying the exposition, in particular the part of Niyogi et al. (2008) concerning the second fundamental form.

The second class of manifolds we consider consists of closed $C^{1,1}$ manifolds $\mathcal {M}$ embedded in $\mathbb {R}^d$. We restrict ourselves to $C^{1,1}$ manifolds because it is known that closed manifolds have positive reach if and only if they are $C^{1,1}$, see Federer (1959, Remarks 4.20 and 4.21) and Scholtes (2013) for a history of this result. Here we do not rely on differential geometry apart from simple concepts such as the tangent space. In fact most proofs can be understood in terms of simple Euclidean geometry. Moreover our proofs are very pictorial. Although the bounds we attain are slightly weaker than the ones we attain using differential geometry, we should note that we have sometimes simplified the exposition at the cost of weakening the bound.

We also prove that the intersection of a $C^{1,1}$ manifold with a ball of radius less than the reach of the manifold is a topological ball. This result is a generalization of previous results. Note that geodesic convexity of a subset does not imply that that the subset is topologically trivial, as the simple example of the circle shows. A sketch of a proof of the result in the $C^2$ case has been given by Boissonnat and Cazals (2001). Attali and Lieutier (2015) proved that the intersection of a set of positive reach and ball of radius less than the reach is contractible. Our topological ball result is stronger, but in a more restricted setting.

*Outline* Section 2 gives the result on metric distortion and geodesic convexity for general sets of positive reach. The third section discusses the variation of tangent spaces, firstly for $C^2$ manifolds and then $C^{1,1}$ manifolds. In the final section we reproof some of the results of the first section using differential geometrical techniques.

## Metric distortion and geodesic convexity

In this section we study distortion and geodesic convexity for general sets of positive reach. We will revisit this topic in Sect. 4 from a smooth viewpoint.

For a closed set $\mathcal {S}\subset \mathbb {R}^d$, $d_{\mathcal {S}}$ denotes the geodesic distance in $\mathcal {S}$, i.e. $d_{\mathcal {S}}(a,b)$ is the infimum of lengths of paths in $\mathcal {S}$ between *a* and *b*. If there is at least one path between *a* and *b* with finite length, then it is known that a minimizing geodesic, i.e. a path with minimal length connecting *a* to *b* exists (see the second paragraph of part III, section 1: “Die Existenz geodätischer Bogen in metrischen Räumen” in Menger 1930).

The next theorem can be read as an alternate definition of the reach, based on metric distortion. Observe that for fixed $|a-b|$, the function $r \mapsto 2 r \arcsin \frac{|a-b|}{ 2 r}$ is decreasing. Note that $2 r \arcsin \frac{|a-b|}{ 2 r}$ is precisely the (geodesic) distance between points *a* and *b* on a circle of radius *r*.

### Theorem 1

If $\mathcal {S}\subset \mathbb {R}^d$ is a closed set, then

$$\begin{aligned} \mathrm {rch}\, \mathcal {S}= \sup \left\{ r > 0, \, \forall a,b \in \mathcal {S}, \, |a-b| < 2 r \Rightarrow d_{\mathcal {S}}(a,b) \le 2 r \arcsin \frac{|a-b|}{ 2 r} \right\} , \end{aligned}$$

where the $\sup$ over the empty set is 0.

The proof of this theorem relies on the the following lemma:

### Lemma 3

Let $\mathcal {S}\subset \mathbb {R}^d$ be a closed set with reach $r= \mathrm {rch}\, \mathcal {S}>0$. For any $a,b\in \mathcal {S}$ such that $|a-b| < 2 r$ one has $d_{\mathcal {S}}(a,b) \le 2 r \arcsin \frac{|a-b|}{ 2 r}.$

The proof of the lemma is technical and takes the remainder of this section. We’ll now prove Theorem 1.

### Proof of Theorem 1

Lemma 3 states that if $r' < \mathrm {rch}\, \mathcal {S}$ then

$$\begin{aligned} \forall a,b \in \mathcal {S}, \, |a-b| < 2 r' \Rightarrow d_{\mathcal {S}}(a,b) \le 2 r '\arcsin \frac{|a-b|}{ 2 r'}. \end{aligned}$$

This gives us

$$\begin{aligned} \sup \left\{ r > 0, \, \forall a,b \in \mathcal {S}, \, |a-b| < 2 r \Rightarrow d_{\mathcal {S}}(a,b) \le 2 r \arcsin \frac{|a-b|}{ 2 r} \right\} \ge \mathrm {rch}\, \mathcal {S}. \end{aligned}$$

If $\mathrm {rch}\, \mathcal {S}= \infty$, i.e. if $\mathcal {S}$ is convex, then for all $a,b \in \mathcal {S}$ and all *r*, we have that $d_\mathcal {S}(a,b)= |a-b| \le 2 r \arcsin \frac{|a-b|}{2r}$ and the theorem holds trivially. We assume now that the medial axis is non empty, i.e. $\mathrm {rch}\, \mathcal {S}< \infty$. Consider $r' > \mathrm {rch}\, \mathcal {S}$. Then by definition of the reach, there exists $x\in \mathbb {R}^d$ in the medial axis of $\mathcal {S}$ and $a,b\in \mathcal {S}, a \ne b$ such that $r' > r_x = d(x, \mathcal {S}) = d(x,a) = d(x,b)$. If for at least one of such pairs $\{a,b\}$ one has $d_{\mathcal {S}}(a,b) = \infty$ then $|a-b| \le 2 r_x < 2 r'$ and:

$$\begin{aligned} \sup \left\{ r > 0, \, \forall a,b \in \mathcal {S}, \, |a-b|< 2 r \Rightarrow d_{\mathcal {S}}(a,b) \le 2 r \arcsin \frac{|a-b|}{ 2 r} \right\} < r' \end{aligned}$$

If not, consider a path $\gamma$ in $\mathcal {S}$ between *a* and *b*: $\gamma (0)=a, \gamma (1) =b$. Because $\gamma ([0,1])$ lies outside the open ball $B(x,r_x)^\circ$, its projection on the closed ball $B(x, r_x )$ cannot increase lengths. It follows that, for any $r \ge r'$:

$$\begin{aligned} d_{\mathcal {S}} (a,b) \ge 2 r_x \arcsin \frac{|a-b|}{ 2 r_x} > 2 r \arcsin \frac{|a-b|}{ 2 r} \end{aligned}$$

which gives, for any $r' > \mathrm {rch}\, \mathcal {S}$,

$$\begin{aligned} \exists a,b \in \mathcal {S}, \forall r \ge r' \quad |a-b| < 2 r \quad \text {and} \quad d_{\mathcal {S}} (a,b) > 2 r \arcsin \frac{|a-b|}{ 2 r}, \end{aligned}$$

and therefore

$$\begin{aligned} \sup \left\{ r > 0, \, \forall a,b \in \mathcal {S}, \, |a-b| < 2 r \Rightarrow d_{\mathcal {S}}(a,b) \le 2 r \arcsin \frac{|a-b|}{ 2 r} \right\} \le r' . \end{aligned}$$

Since this holds for any $r'> \mathrm {rch}\, \mathcal {S}$ we get:

$$\begin{aligned} \sup \left\{ r > 0, \, \forall a,b \in \mathcal {S}, \, |a-b| < 2 r \Rightarrow d_{\mathcal {S}}(a,b) \le 2 r \arcsin \frac{|a-b|}{ 2 r} \right\} \le \mathrm {rch}\, \mathcal {S}. \end{aligned}$$

$$\square $$

We now remind ourselves that a set is geodesically convex if the minimizing geodesic between any two points of the set is itself contained in the set. With this definition we can give the following result:

### Corollary 1

Let $\mathcal {S}\subset \mathbb {R}^d$ be a closed set with positive reach $r= \mathrm {rch}\, \mathcal {S}>0$. Then, for any $r' < \mathrm {rch}\, \mathcal {S}$ and any $x \in \mathbb {R}^d$, if $B(x,r')$ is the closed ball centered at *x* with radius $r'$, then $\mathcal {S}\cap B(x,r')$ is geodesically convex in $\mathcal {S}$.

### Proof

First it follows from the theorem that if $a,b \in \mathcal {S}\cap B(x,r')$, then $d_{\mathcal {S}}(a,b) < \infty$ which means that there exists a path of finite length in $\mathcal {S}$ between *a* and *b*. From Menger (1930) there is at least one minimizing geodesic in $\mathcal {S}$ between *a* and *b*.

For a contradiction assume that such a geodesic $\gamma$ goes outside $B(x,r')$. In other words there is at least one non empty open interval $(t_1,t_2)$ such that $\gamma (t_1), \gamma (t_2) \in \partial B(x,r')$ and $\gamma ( (t_1,t_2)) \cap B(x,r') = \emptyset$. But then, since the projection on the ball $B(x,r')$ reduces lengths, one has:

$$\begin{aligned} d_{\mathcal {S}}(\gamma (t_1), \gamma (t_2)) > 2 r' \arcsin \frac{|\gamma (t_1)-\gamma (t_2)|}{ 2 r'} , \end{aligned}$$

a contradiction with the theorem. $\square$

We emphasize that the question of convexity has not been considered before.

### Projection of the middle point

Sections 2.1 and 2.2 are devoted to the proof of Lemma 3, which is the technical part of the proof of Theorem 1.

**Fig. 1:**
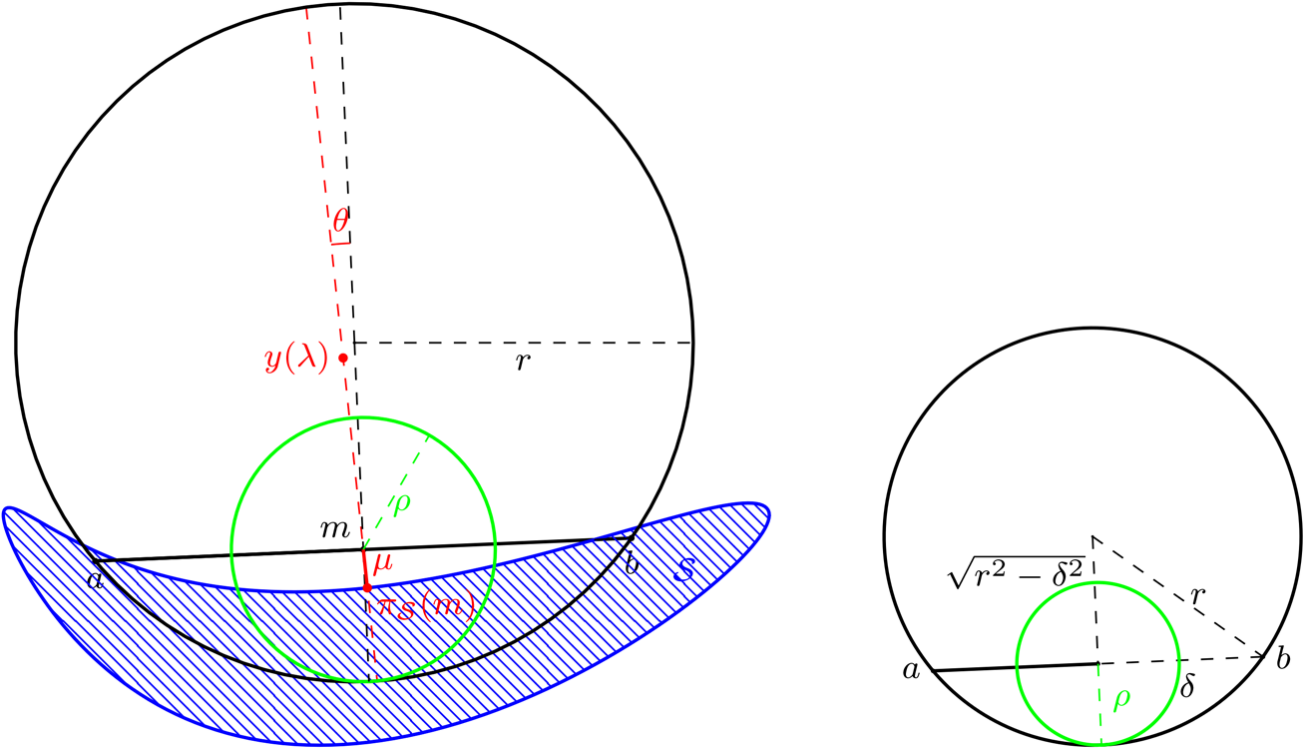
On the left the projection $\pi _{\mathcal {S}} (m)$ is contained in the disk of center *m* and radius $\rho$. The notation used in the proof of Lemma 1 is also added. From the right figure it is easy to deduce that $\rho = r - \sqrt{r^2 - \delta ^2}$

For a closed set $\mathcal {S}\subset \mathbb {R}^d$ with positive reach $r= \mathrm {rch}\, \mathcal {S}>0$ and a point $m\in \mathbb {R}^d$ with $d(m,\mathcal {S}) < r$, $\pi _{\mathcal {S}} (m)$ denotes the projection of *m* on $\mathcal {S}$ as depicted on Fig. 1 on the left.

#### Lemma 1

Let $\mathcal {S}\subset \mathbb {R}^d$ be a closed set with reach $r= \mathrm {rch}\, \mathcal {S}>0$. For $a,b\in \mathcal {S}$ such that $\delta = \frac{|a-b|}{2} < r$ and $m = \frac{a+b}{2}$ one has $|\pi _{\mathcal {S}} (m) - m | \le \rho ,$ with $\rho = r - \sqrt{r^2 - \delta ^2}.$

The disk of center *m* and radius $\rho$ appears in green in Fig. 1 left and right.

#### Proof

We shall now use a consequence of Theorem 4.8 of Federer (1959). In the following section we shall discuss this result for the manifold setting, where it generalizes the tubular neighbourhood results for $C^2$ manifolds from differential geometry and differential topology. For the moment we restrict ourselves to the following: If $\pi _{\mathcal {S}} (m) \ne m$ claim (12) in Theorem 4.8 of Federer (1959) gives us:

$$\begin{aligned} \forall \lambda \in [0,r), \, \pi _{\mathcal {S}} \left( \pi _{\mathcal {S}} (m) + \lambda \frac{m - \pi _{\mathcal {S}} (m) }{|m - \pi _{\mathcal {S}} (m) | } \right) = \pi _{\mathcal {S}} (m) , \end{aligned}$$

which means that for $\lambda \in [0,r)$:

$$\begin{aligned} y(\lambda ) = \pi _{\mathcal {S}} (m) + \lambda \frac{m - \pi _{\mathcal {S}} (m) }{|m - \pi _{\mathcal {S}} (m) | } \end{aligned}$$

is closer to $\pi _{\mathcal {S}} (m)$ than both to *a* and to *b* (see Fig. 1).

Without loss of generality we assume that $| a - \pi _{\mathcal {S}} (m) | \ge | b - \pi _{\mathcal {S}} (m) |$. We denote $\mu = | \pi _{\mathcal {S}} (m) - m |$ and want to prove that $\mu \le \rho$.

In the plane spanned by $a,b, \pi _{\mathcal {S}} (m)$ we consider the following frame $(m, \frac{a-m}{|a-m|}, \tau )$, where *m* denotes the origin, $\tau$ is a unit vector orthogonal to $a-m$ and such that $\langle \tau , \pi _{\mathcal {S}} (m) - m \rangle \le 0$.

For some $\theta \in [0, \pi /2]$, the coordinates of $\pi _{\mathcal {S}} (m)$ in the frame are $(- \mu \sin \theta , -\mu \cos \theta )$. The coordinate of *a* are $(\delta , 0)$ and the coordinates of $y(\lambda )$ are, as shown in Fig. 1, $((\lambda -\mu ) \sin \theta , (\lambda -\mu ) \cos \theta ).$ Since $y(\lambda )$ is closer to $\pi _{\mathcal {S}} (m)$ than to *a*, one has

$$\begin{aligned} \forall \lambda \in [0,r), \quad \left( \delta - (\lambda -\mu ) \sin \theta \right) ^2 + (\lambda - \mu )^2 \cos ^2 \theta > \lambda ^2. \end{aligned}$$

This is a degree 2 inequality in $\mu$. One gets, for any $\lambda \in [0,r)$, if $\varDelta \ge 0$,

$$\begin{aligned} \mu \notin \left[ (\lambda - \delta \sin \theta ) - \sqrt{\varDelta }, \; (\lambda - \delta \sin \theta ) + \sqrt{\varDelta } \right] , \end{aligned}$$

with $\varDelta = (\lambda - \delta \sin \theta )^2 - (\delta ^2 - 2 \delta \lambda \sin \theta ) = \lambda ^2 -\delta ^2 +(\delta \sin \theta )^2.$ For $\lambda \ge \delta$ one has $\varDelta \ge \lambda ^2 -\delta ^2$. Therefore: $(\lambda - \delta \sin \theta ) - \sqrt{\varDelta } \le \lambda - \sqrt{\lambda ^2 - \delta ^2}$ and since $\lambda \mapsto \lambda - \sqrt{\lambda ^2 - \delta ^2}$ is continuous, one has:

$$\begin{aligned} \inf _{\lambda < r} \left\{ (\lambda - \delta \sin \theta ) - \sqrt{\varDelta } \right\} \le r - \sqrt{r^2 - \delta ^2}= \rho , \end{aligned}$$

also, when $\lambda \ge \delta$ one has $\sqrt{\varDelta } \ge \delta \sin \theta$ and $(\lambda - \delta \sin \theta ) + \sqrt{\varDelta } \ge \delta .$ Since $\mu \le d(m,a) = \delta$, one finds that $\mu \le \rho$. $\square$

The following simple geometric Lemma is used in the next section.

#### Lemma 2

Consider a circle $\tilde{C}$ of radius *r* and two points $a,b \in \tilde{C}$ with $|a-b|/2 = \delta < r.$ Define the middle point $m=\frac{a+b}{2}$ and consider a point *p* such that $|p-m | \le \rho = r - \sqrt{r^2 - \delta ^2}.$ Denote $\tilde{C}_ {a,b}$ the shortest of the arcs of the circle in $\tilde{C}$ bounded by *a* and *b*. Define $\tilde{p} \in \tilde{C}_ {a,b}$ as the unique point in $\tilde{C}_ {a,b}$ such that $\frac{| a - \tilde{p} | }{| b- \tilde{p} | }= \frac{| a-p| }{| b -p | }$, then we have $| a-p | \le | a-\tilde{p} |$ and $| b-p | \le | b -\tilde{p} |$.

#### Proof

Since $\rho <r$, one has $| b-p | \ge \delta - \rho >0$ and the quotient is well defined. Because $|p-m | \le \rho$, *p* belongs to both disks with radius *r* with *a* and *b* on their boundary. This can be expressed through angles comparison as $\psi = \angle apb \ge \angle a\tilde{p}b = \tilde{\psi } \ge \pi /2.$ If we denote $\tau = \frac{| a-p| }{| b-p | }$ one has

$$\begin{aligned} (a-b)^2= & {} \left( (a-p) + (p-b) \right) ^2 \\= & {} |a-p|^2 + |b-p|^2 - 2 |a-p| |b-p| \cos \psi \\= & {} | b-p |^2 \left( 1 + \tau ^2 - 2 \tau \cos \psi \right) . \end{aligned}$$

Similarly,

$$\begin{aligned} (a-b)^2 = | b- \tilde{p} |^2 \left( 1 + \tau ^2 - 2 \tau \cos \tilde{\psi } \right) , \end{aligned}$$

so that

$$\begin{aligned} = | b- \tilde{p} |^2 \left( 1 + \tau ^2 - 2 \tau \cos \tilde{\psi } \right) = | b-p |^2 \left( 1 + \tau ^2 - 2 \tau \cos \psi \right) . \end{aligned}$$

But $$\psi \ge \tilde{\psi } \ge \pi /2$$ gives

$$\begin{aligned} 1 + \tau ^2 - 2 \tau \cos \tilde{\psi } \le 1 + \tau ^2 - 2 \tau \cos \psi , \end{aligned}$$

and we get $| b -\tilde{p} | \ge | b -p |$ and $| a -\tilde{p} | = \frac{| b -\tilde{p}| }{ | b-p |} |a-p| \ge |a-p|$. $\square$

### Metric distortion

In this section we establish an upper bound on geodesic lengths through the iterative construction of a sequence of paths.

#### Lemma 3

Let $\mathcal {S}\subset \mathbb {R}^d$ be a closed set with reach $r= \mathrm {rch}\, \mathcal {S}>0$. For any $a,b\in \mathcal {S}$ such that $|a-b| < 2 r$ one has $d_{\mathcal {S}}(a,b) \le 2 r \arcsin \frac{|a-b|}{ 2 r}.$

#### Proof

**Fig. 2:**
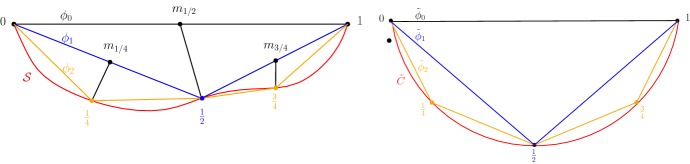
Left: $$\phi _0,\phi _1, \phi _2$$. Right: $$\tilde{\phi }_0,\tilde{\phi }_1, \tilde{\phi }_2$$

We build two sequences of PL-functions (see Fig. 2). For $i\in \mathbb {N}$, $\phi _i : [0,1] \rightarrow \mathbb {R}^d$ and $\tilde{\phi }_i : [0,1] \rightarrow \mathbb {R}^2$ are defined as follows.

First we define $\phi _0(t) = a + t (b-a).$ Denote $m=\frac{a+b}{2}$ the middle point of [*a*, *b*]. Since $d(m, \mathcal {S}) \le d(m,a) = \delta < r$, the point $p=\pi _{\mathcal {S}} (m)$ is well defined. Secondly, we define

$$\begin{aligned} \phi _1(t) = {\left\{ \begin{array}{ll} a + 2t (p-a) \, &{} \quad \text {if} \quad t \le 1/2\\ p + (2t-1) (b-p) \, &{} \quad \text {if} \quad t \ge 1/2. \end{array}\right. } \end{aligned}$$

as depicted in Fig. 2 on the left.

From Lemma 1, one has $| p-m | \le \rho = r - \sqrt{r^2 - \delta ^2} < r$ and thus

$$\begin{aligned} \min \left( | a-p |, |b-p| \right)&\ge \delta - \rho >0&\max \left( | a-p|, |b-p| \right)&\le \delta + \rho \end{aligned}$$

We also fix a circle $\tilde{C}$ in $\mathbb {R}^2$ with radius *r* and we consider $\tilde{a}, \tilde{b} \in \mathbb {R}^2$ such that $\tilde{a}, \tilde{b} \in \tilde{C}$ and $|\tilde{a} - \tilde{b}| = |a-b|$ and we define $\tilde{\phi }_0(t) = \tilde{a} + t (\tilde{b}-\tilde{a}).$ Denote by $\tilde{C}_ {\tilde{a}, \tilde{b}}$ the shortest of the two arcs of $\tilde{C}$ bounded by $\tilde{a}, \tilde{b}$ and $\tilde{p}$ as constructed in Lemma 2 i.e. $\tilde{p}\in \tilde{C}_ {\tilde{a}, \tilde{b}}$ such that $\frac{| \tilde{p} - \tilde{a} | }{| \tilde{p} - \tilde{b} | }= \frac{| p-a| }{| p-b | },$ as shown in Fig. 2 on the right, and define

$$\begin{aligned} \tilde{\phi }_1(t) = {\left\{ \begin{array}{ll} \tilde{a} + 2t ( \tilde{p}- \tilde{a}) \, &{} \quad \text {if} \quad t \le 1/2\\ \tilde{p} + (2t-1) ( \tilde{b}- \tilde{p}) \, &{} \quad \text {if} \quad t \ge 1/2. \end{array}\right. } \end{aligned}$$

Applying Lemma 2 we get $| a-p | \le | \tilde{a} -\tilde{p} |$, $| b-p | \le | \tilde{b} -\tilde{p} |,$ and

$$\begin{aligned} \text {length} (\phi _1) = | a-p | + | b-p| \le | \tilde{a} -\tilde{p} | + | \tilde{b} -\tilde{p} | = \text {length} (\tilde{\phi }_1). \end{aligned}$$

For $i \ge 2$, $\phi _i$ and $\tilde{\phi }_i$ are PL functions with $2^i$ intervals. For $k\in \mathbb {N}$, $0 \le k \le 2^i$, $\phi _i (k/ 2^i) \in \mathcal {S}$, $\tilde{\phi }_i (k/ 2^i) \in \tilde{C}_ {\tilde{a}, \tilde{b}}$ are defined by applying to each of the $2^{i-1}$ segments of $\phi _{i-1}([0,1])$ and $\tilde{\phi }_{i-1}([0,1])$ the same subdivision process used when defining $\phi _{1}$ and $\tilde{\phi }_{1}$.

If *k* is even we set $\phi _i(k/ 2^i) = \phi _{i-1}(k/ 2^i)$ and $\tilde{\phi _i}(k/ 2^i) = \tilde{\phi }_{i-1}(k/ 2^i)$.

If *k* is odd define:

$$\begin{aligned} m_{k/ 2^i} = \frac{ \phi _{i}((k-1)/ 2^i) + \phi _{i}((k+1)/ 2^i) }{ 2} \quad \text {and} \quad \phi _i(k/ 2^i) = \pi _{\mathcal {S}} \left( m_{k/ 2^i} \right) . \end{aligned}$$

Note that $m_{1/2}$ corresponds to *m* defined above.

Let $\tilde{\phi _i}(k/ 2^i) \in \tilde{C}_ {\tilde{\phi }_{i-1}((k-1)/ 2^i) , \tilde{\phi }_{i-1}((k+1)/ 2^i) } \subset \tilde{C}_ {\tilde{a}, \tilde{b}}$ be such that:

$$\begin{aligned} \frac{| \tilde{\phi _i}(k/ 2^i) - \tilde{\phi }_{i-1}((k-1)/ 2^i) | }{| \tilde{\phi _i}(k/ 2^i) - \tilde{\phi }_{i-1}((k+1)/ 2^i) | }= \frac{| \phi _i(k/ 2^i)-\phi _{i-1}((k-1)/ 2^i)| }{| \phi _i(k/ 2^i)- \phi _{i-1}((k+1)/ 2^i) | }. \end{aligned}$$

Figure 2 left shows the curves $\phi _1$ and $\phi _2$ in blue and yellow respectively.

Applying Lemma 2, since by induction,

$$\begin{aligned} \left| \phi _{i-1}((k+1)/ 2^{i-1}) - \phi _{i-1}(k/ 2^{i-1}) \right| \le \left| \tilde{\phi }_{i-1}((k+1)/ 2^{i-1}) - \tilde{\phi }_{i-1}(k/ 2^{i-1}) \right| \end{aligned}$$

we get that for $i \in \mathbb {N}$ and $p=0,\ldots , 2^i -1$:

$$\begin{aligned} | \phi _i((k+1) / 2^i) - \phi _i(k/ 2^i) | \le | \tilde{\phi }_i((k+1) / 2^i) - \tilde{\phi }_i(k/ 2^i) | , \end{aligned}$$

and therefore:

1 $$\begin{aligned} \text {length} (\phi _i)= & {} \sum _{k=0}^{2^i -1}| \phi _i((k+1) / 2^i) - \phi _i(k/ 2^i) | \nonumber \\\le & {} \sum _{k=0}^{2^i -1}| \tilde{\phi }_i((k+1) / 2^i) - \tilde{\phi }_i(k/ 2^i) | \nonumber \\= & {} \text {length} (\tilde{\phi }_i) \le \text {length} (\tilde{C}_ {\tilde{a}, \tilde{b}}) = 2 r \arcsin \frac{|a-b|}{ 2 r}. \end{aligned}$$

We study now the behavior of the sequence $\phi _i, i \in \mathbb {N}$. Define $\delta _0 = \delta$ and $\rho _0 = \rho$. Further define $\delta _i$ as

$$\begin{aligned} \delta _i = \frac{1}{2} \max _{0\le k\le 2^i -1} | \phi _i((k+1)/2^i ) - \phi _i(k/2^i ) |. \end{aligned}$$

i.e. half the max of lengths of all segments of $\phi _i([0,1])$ and $\rho _i = r - \sqrt{r^2 - \delta _i^2 }$. We make the following assertion: $\square$

#### Claim

2 $$\begin{aligned} \lim _{i\rightarrow \infty } \delta _i = 0. \end{aligned}$$

#### Proof of the claim

Thanks to the definitions of $\delta _i$ and $\rho _i$, one has for $i\ge 1$

3 $$\begin{aligned} \delta _i \le \frac{1}{2} (\delta _{i-1} + \rho _{i-1}) = \frac{1}{2} \left( 1 + \frac{\rho _{i-1}}{ \delta _{i-1}} \right) \delta _{i-1}. \end{aligned}$$

Moreover for any $i\in \mathbb {N}$,

4 $$\begin{aligned} \frac{\rho _{i}}{ \delta _{i}} = \frac{r}{\delta _{i}} - \sqrt{ \left( \frac{r}{ \delta _{i}} \right) ^2 -1 } = \frac{1}{ \frac{r}{ \delta _{i} } + \sqrt{ \left( \frac{ r}{ \delta _{i}} \right) ^2 -1 } } \le \frac{ \delta _{i} }{r}. \end{aligned}$$

Equations (3) and (4) give:

5 $$\begin{aligned} \delta _i \le \frac{1}{2} \left( 1 + \frac{ \delta _{i-1}}{r} \right) \delta _{i-1}. \end{aligned}$$

Since

$$\begin{aligned} \frac{ \delta _{0}}{r} = \frac{ \delta }{r} < 1, \end{aligned}$$

(5) allows the induction

$$\begin{aligned} \frac{ \delta _{i}}{r}< 1 \Rightarrow \frac{ \delta _{i+1}}{r}< \frac{ \delta _{i}}{r} <1. \end{aligned}$$

We get that the sequence $\left( \delta _{i}\right) _{i\in \mathbb {N}}$ is decreasing and $\frac{ \delta _{i}}{r} \le \frac{ \delta }{r}$. Replacing and iterating in (5) gives

$$\begin{aligned} \delta _i \le \left( \frac{1}{2} \left( 1 + \frac{ \delta _0}{r} \right) \right) ^i \delta _0. \end{aligned}$$

Since $\frac{1}{2} \left( 1 + \frac{ \delta _0}{r} \right) <1$ we see that $\delta _i$ decreases faster than a geometric sequence, in particular:

6 $$\begin{aligned} \lim _{i\rightarrow \infty } \delta _i = 0. \end{aligned}$$

$$\square $$

Since for any $i\ge 0$ and $t\in [0,1]$, $d(\phi (t), \mathcal {S}) \le \delta _i$ and $\delta _i < \mathrm {rch}\, \mathcal {S}$ the curves $\pi _{\mathcal {S}} \circ \phi _i$, (projections of $\phi _i$ on $\mathcal {S}$) are well defined, with $\pi _{\mathcal {S}} \circ \phi _i : [0,1] \rightarrow \mathcal {S}$, $\pi _{\mathcal {S}} \circ \phi _i (0) =a$ and $\pi _{\mathcal {S}} \circ \phi _i(1) =b$.

Claim (8) in Theorem 4.8 of Federer (1959) states that for $\mu < r= \mathrm {rch}\mathcal {S}$ the restriction of $\pi _{\mathcal {S}}$ to the $\mu$-tubular neighbourhood $\mathcal {S}^\mu$ is $\frac{\mathrm {rch}\mathcal {S}}{\mathrm {rch}\mathcal {S}- \mu }$-Lipschitz. This together with (1) above gives us an upper bound on the lengths of curves $\pi _{\mathcal {S}} \circ \phi _i$:

$$\begin{aligned} \text {length} (\pi _{\mathcal {S}} \circ \phi _i) \le \frac{\mathrm {rch}\, \mathcal {S}}{\mathrm {rch}\, \mathcal {S}- \delta _i} \text {length} (\phi _i) \le \frac{\mathrm {rch}\, \mathcal {S}}{\mathrm {rch}\, \mathcal {S}- \delta _i} 2 r \arcsin \frac{|a-b|}{ 2 r} \end{aligned}$$

This together with (2) yields $d_{\mathcal {S}}(a,b) \le 2 r \arcsin \frac{|a-b|}{ 2 r}$. $\square$

## Variation of tangent spaces

In this section we shall bound the variation of tangent spaces in the $C^2$ setting, and then generalize to the $C^{1,1}$ setting. For this generalization we need a topological result, which will be presented in Sect. 3.2.

### Bounds for $C^2$ submanifolds

We shall be using the following result, Theorem 4.8(12) of Federer (1959):

#### Theorem 2

(Federer’s tubular neighbourhoods) Consider a manifold $\mathcal {M}$ of positive reach $\mathrm {rch}(\mathcal {M})$ and a non-negative real number *r* smaller than the reach. Let $B_{N_p\mathcal {M}} (r)$, be the ball of radius *r* centred at *p* in the normal space $N_p\mathcal {M}\subset \mathbb {R}^d$. For every point $x \in B_{N_p\mathcal {M}} (r)$, $\pi _{\mathcal {M}}(x)=p$.

The fact that such a tubular neighbourhood exists is non-trivial, even if we take $r=\epsilon$. From Theorem 2 we immediately see that:

#### Corollary 2

Let $\mathcal {M}$ be a submanifold of $\mathbb {R}^d$ and $p\in \mathcal {M}$. Any open ball *B*(*c*, *r*) that is tangent to $\mathcal {M}$ at *p* and whose radius *r* satisfies $r\le \mathrm {rch}(\mathcal {M})$ does not intersect $\mathcal {M}$.

#### Proof

Let $r < \mathrm {rch}(\mathcal {M})$. Suppose that the intersection of $\mathcal {M}$ and the open ball is not empty, then $\pi _{\mathcal {M}}(c)\ne p$ contradicting Federer’s tubular neighbourhood theorem. Now suppose that the open ball of radius $\mathrm {rch}(\mathcal {M})$ contains a point *q*. Then there exists an $r' <\mathrm {rch}(\mathcal {M})$ and a ball of radius $r'$ tangent to $\mathcal {M}$ at *p* such that *q* lies inside this ball. This again gives a contradiction. $\square$

Here we prove the main result for $C^2$ manifolds. Our exposition is the result of straightforwardly combining the work of Niyogi et al. (2008), and the two dimensional analysis of Attali et al. (2007) with some observations concerning the reach.

We start with the following simple observation:

#### Lemma 4

Let $\gamma (t)$ be a geodesic parametrized according to arc length on $\mathcal {M}\subset \mathbb {R}^d$, then $|\ddot{\gamma } | \le 1/ \mathrm {rch}(\mathcal {M})$, where we use Newton’s notation, that is we write $\ddot{\gamma }$ for the second derivative of $\gamma$ with respect to *t*.

#### Proof

Because $\gamma (t)$ is a geodesic, $\ddot{\gamma }(t)$ is normal to $\mathcal {M}$ at $\gamma (t)$. Now consider the sphere of radius $\mathrm {rch}(\mathcal {M})$ tangent to $\mathcal {M}$ at $\gamma (t)$, whose centre lies on the line $\{ \gamma (t)+ \lambda \ddot{\gamma } \mid \lambda \in \mathbb {R} \}$. If now $|\ddot{\gamma } |$ were larger than $1/ \mathrm {rch}(\mathcal {M})$, the geodesic $\gamma$ would enter the tangent sphere, which would contradict Corollary 2. $\square$

Note that $|\ddot{\gamma } |$ is the normal curvature, because $\gamma$ is a geodesic. Using the terminology of Niyogi et al. (2008, Section 6), Lemma 4 can also be formulated as follows: $1/ \mathrm {rch}(\mathcal {M})$ bounds the principal curvatures in the normal direction $\nu$, for any unit normal vector $\nu \in N_p \mathcal {M}$. In particular, $1/ \mathrm {rch}(\mathcal {M})$ also bounds the principal curvatures if $\mathcal {M}$ has codimension 1.

We now have the following, which is a straightforward extension of an observation in Attali et al. (2007) to general dimension:

#### Lemma 5

Let $\gamma (t)$ be a geodesic parametrized according to arc length, with $t\in [0,\ell ]$ on $\mathcal {M}\subset \mathbb {R}^d$, then:

$$\begin{aligned} \angle \dot{\gamma }(0) \dot{\gamma }(\ell ) \le \frac{d_\mathcal {M}(\gamma (0),\gamma (\ell )) }{\mathrm {rch}(\mathcal {M})} . \end{aligned}$$

#### Proof

Because $\gamma$ is parametrized according to arc length $| \dot{\gamma } |=1$ and $\dot{\gamma }(t)$ can be seen as a curve on the sphere $\mathbb {S}^{d-1}$. Moreover $\ddot{\gamma }$ can be seen as tangent to this sphere. The angle between two tangent vectors $\dot{\gamma }(0)$ and $\dot{\gamma }(\ell )$ equals the geodesic distance on the sphere. The geodesic distance between any two points is smaller or equal to the length of any curve connecting these points, and $\{\dot{\gamma }(t) \mid t \in [0,\ell ] \}$ is such a curve. We therefore have

7 $$\begin{aligned} \angle \dot{\gamma }(0) \dot{\gamma }(\ell ) \le \int _{0}^{\ell } \left| \frac{d}{dt} \dot{\gamma } \right| \mathrm {d}t =\int _{0}^{\ell } | \ddot{\gamma } | \mathrm {d}t \le \frac{\ell }{\mathrm {rch}(\mathcal {M})} \le \frac{d_\mathcal {M}(\gamma (0),\gamma (\ell )) }{\mathrm {rch}(\mathcal {M})}, \end{aligned}$$

where we used Lemma 4. $\square$

We can now turn our attention to the variation of tangent spaces. Here we mainly follow Niyogi et al. (2008), but use one useful observation of Attali et al. (2007). We shall be using the second fundamental form, which we assume the reader to be familiar with. We refer to do Carmo (1992) as a standard reference.

The second fundamental form  has the geometric interpretation of the normal part of the covariant derivative, where we assume now that *u*, *v* are vector fields. In particular , where $\bar{\nabla }$ is the connection in the ambient space, in this case Euclidean space, and $\nabla$ the connection with respect to the induced metric on the manifold $\mathcal {M}$. The second fundamental form  is a symmetric bi-linear form, see for example Section 6.2 of do Carmo (1992) for a proof. This means that we only need to consider vectors in the tangent space and not vector fields, when we consider .

We can now restrict our attention to *u*, *v* lying on the unit sphere $\mathbb {S}^{n-1}_{T_p\mathcal {M}}$ (of codimension one in $T_p\mathcal {M}$) in the tangent space and ask for which of these vectors  is maximized. Let us assume that the  for which the maximum^1^ is attained lies in the direction of $\eta \in N_p \mathcal {M}$ where $\eta$ is assumed to have unit length.

We can now identify , with a symmetric matrix. Because of this , with $u,v\in \mathbb {S}^{n-1}_{T_p\mathcal {M}}$, attains its maximum for *u*, *v* both lying in the direction of the unit eigenvector *w* of  with the largest^2^ eigenvalue. In other words the maximum is assumed for $u=v=w$. Let us now consider a geodesic $\gamma _w$ on $\mathcal {M}$ parametrized by arclength such that $\gamma _w(0)=p$ and $\dot{\gamma }_w(0)=w$. Now, because $\gamma _w$ is a geodesic and the ambient space is Euclidean,Due to Lemma 4 and by definition of the maximum, we now see that , for all *u*, *v* of length one.

Having discussed the second fundamental form, we can give the following lemma:

#### Lemma 6

Let $$p,q \in \mathcal {M}$$, then

$$\begin{aligned} \angle (T_p\mathcal {M}, T_q \mathcal {M}) \le \frac{d_\mathcal {M}(p,q)}{\mathrm {rch}(\mathcal {M})}. \end{aligned}$$

#### Proof

Let $\gamma$ be a geodesic connecting *p* and *q*, parametrized by arc length. We consider an arbitrary unit vector *u* and parallel transport this unit vector along $\gamma$, getting the unit vectors *u*(*t*) in the tangent spaces $T_{\gamma (t)}\mathcal {M}$. The maximal angle between *u*(0) and $u(\ell )$, for all *u* bounds the angle between $T_p\mathcal {M}$ and $T_q \mathcal {M}$. Nowwhere we used that *u*(*t*) is parallel and thus by definition $\nabla _{\dot{\gamma }} u(t) =0$. So using our discussion above $|\frac{d u}{dt}| \le 1/ \mathrm {rch}(\mathcal {M})$. Now we note that, similarly to what we have seen in the proof of Lemma 5, *u*(*t*) can be seen as a curve on the sphere and thus $\angle (u(0), u(\ell )) \le \int _0^{\ell } |\frac{d u}{dt}| \mathrm {d}t \le \ell / \mathrm {rch}(\mathcal {M})$. $\square$

This bound is tight as it is attained for a sphere.

Combining Theorem 1 and Lemma 6 we find that

#### Corollary 3

$$\begin{aligned} \sin \left( \frac{\angle (T_p\mathcal {M}, T_q \mathcal {M})}{2} \right) \le \frac{|p-q|}{2 \, \mathrm {rch}(\mathcal {M})} . \end{aligned}$$

#### Proof

Lemma 6 gives

$$\begin{aligned} \sin \left( \frac{\angle (T_p\mathcal {M},T_q \mathcal {M}) }{2} \right) \le \sin \left( \frac{d_\mathcal {M}(p,q)}{2\, \mathrm {rch}(\mathcal {M})} \right) \end{aligned}$$

and Theorems 1 yields

$$\begin{aligned} \sin \left( \frac{d_\mathcal {M}(p,q)}{2\, \mathrm {rch}(\mathcal {M})} \right) \le \frac{|p-q|}{2 \, \mathrm {rch}(\mathcal {M})} . \end{aligned}$$

The result now follows. Note that the statement holds trivially if $|p-q|\ge 2 \, \mathrm {rch}(\mathcal {M})$. $\square$

With the bound on the angles between the tangent spaces it is not difficult to prove that the projection map onto the tangent space is locally a diffeomorphism, as has been done in Niyogi et al. (2008). Although the results were given in terms of the (global) reach to simplify the exposition, the results can be easily formulated in terms of the local feature size.

### A topological result

We shall now give a full proof of a variant of a statement by Boissonnat and Cazals (2001, Proposition 12) in the more general $C^{1,1}$ setting:

#### Proposition 1

If *B* is a closed ball of radius strictly less than the reach that intersects a $C^{1,1}$ manifold $\mathcal {M}$, then $B \cap \mathcal {M}$ is a topological ball. Here we include points (balls of dimension/radius 0).

Note that this result is not implied by Corollary 1, because subspaces can be geodesically convex without being topological disks, think for example of the equator of the sphere.

The proof uses some results from topology, namely variants of Milnor (1969, Theorem 3.2 and Theorem 3.1):

#### Lemma 7

Consider the distance function from *c*: $d_c:\mathbb {R}^d\rightarrow \mathbb {R}, d_c(x)=| x-c|$ restricted to $\mathcal {M}$. Let $a= d_c(x' )$ and $b=r$ and suppose that the set $d_c^{-1} [a,b]$, consisting of all $p\in \mathcal {M}$ with $a\le d_c(p) \le b$, contains no critical points of $d_c$ (that is, no point *q* of $\mathcal {M}$ where *B*(*c*, *q*) is tangent to $\mathcal {M}$). Then $\mathcal {M}^a=\{ x\in \mathcal {M}, d_c\le a\} = \mathcal {M}\cap B(c,a)$ is homeomorphic (if $d_c$ is $C^{1,1}$) to $\mathcal {M}^b=\{ x\in \mathcal {M}, d_c\le b\}$. Furthermore $M^a$ is a deformation retract of $M^b$.

#### Proof

The key change compared to original statement by Milnor, which is in the $C^2$ setting, is the passing from a diffeomorphism to a homeomorphism. This lemma is true because of the following: The proof of Theorem 3.1 of Milnor (1969) mentions the assumption that the function (in this case $d_c$) is smooth, however in the proof relies on using gradient flow, that is solving a differential equation. Thanks to Picard–Lindelöf theorem, see Coddington and Levinson (1987, Theorem 3.1), we know that the initial value problem $\dot{x}=g(x)$, where $\dot{x}$ denotes the derivative with respect to time, has a unique continuous solution if *g* is Lipschitz. In the proof presented by Milnor, *g* is the gradient of a (Morse) function (in this case the distance function). This implies that it suffices that the gradient of the distance function is Lipschitz, or equivalently that the function itself is $C^{1,1}$. Because the gradient flow is only continuous in this Lipschitz setting we find a homeomorphism in the $C^{1,1}$ setting, instead of the diffeomorphism as in the $C^2$ case. $\square$

#### Lemma 8

Let $d_c|_\mathcal {M}$ be the $C^{1,1}$ function on $\mathcal {M}$ defined, as in Lemma 7, as the restriction to $\mathcal {M}$ of $d_c:\mathbb {R}^d\rightarrow \mathbb {R}, d_c(x)=| x-c|$. Assume that *y* is a global isolated minimum of $d_c|_\mathcal {M}$ and let $r_c$ be the second critical value of $d_c|_\mathcal {M}$. Then for all $0<\eta < r_c-|c-y|$, $\mathcal {M}^{r_c- \eta }$ is a topological ball.

#### Proof

Due to Lemma 7, in particular the deformation retract, we have that $\mathcal {M}^{r_c- \eta }{\setminus } \{ y\}$ is homeomorphic to $(0,1] \times (d_c|_\mathcal {M}) ^{-1} (r_c- \eta )$, for all $0<\eta < r_c-|c-y|$. This gives that $\mathcal {M}^{r_c- \eta }$ is homeomorphic to the cone of $(d_c|_\mathcal {M}) ^{-1} (r_c- \eta )$ with the point *y* as its tip. Because $\mathcal {M}^{r_c- \eta }$ is a $C^{1,1}$ manifold with boundary and *y* does not lie on its boundary we have the following: Firstly, $(d_c|_\mathcal {M}) ^{-1} (r_c- \eta )$ is a $C^{1}$ manifold and it can be triangulated, see Palais (1963, section 7) and Whitehead (1940) respectively, giving a triangulation of the cone by taking the join of each simplex in the triangulation of $(d_c|_\mathcal {M}) ^{-1} (r_c- \eta )$ with *y*. We can now use the following definition and result from topology (Zeeman 1963, Chapter 3):

#### Definition 1

(*Combinatorial manifold*) A complex *K* is called a combinatorial *n*-manifold if the link (the boundary of the star) of each vertex is an $(n-1)$-sphere or an $(n-1)$-ball.

#### Lemma 9

(Zeeman 1963, Lemma 9 of Chapter 3) Suppose that $|K|=\mathcal {M}$. Then *K* is a combinatorial manifold if and only if $\mathcal {M}$ is a PL-manifold.

Because $(d_c|_\mathcal {M}) ^{-1} (r_c- \eta )$ is the link of *y*, $(d_c|_\mathcal {M}) ^{-1} (r_c- \eta )$ is a sphere and $\mathcal {M}^{r_c- \eta }$ a ball. $\square$

**Fig. 3:**
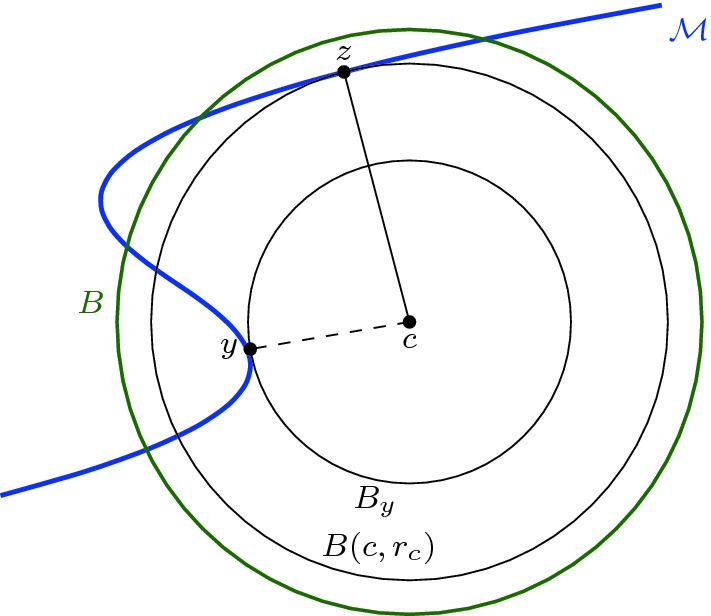
For the proof of Proposition 1

#### Proof of Proposition 1

Write *r* for the radius of *B* and *c* for its center. The result is trivial if *c* belongs to the closure of the medial axis of $\mathcal {M}$, because then the intersection $B\cap \mathcal {M}$ is empty. Therefore assume that $c\not \in \text {axis} (\mathcal {M})$.

Let *y* be the (unique) point of $\mathcal {M}$ closest to *c*. We denote by $B_y$ the closed ball centered at *c* with radius $| c-y|$ (see Fig. 3). By Corollary 2, the interior of $B_y$ does not intersect $\mathcal {M}$ and $B_y \cap \mathcal {M}=\{ y\}$. This means that the conditions of Lemma 8 are satisfied and $B(c, r_c- \eta ) \cap \mathcal {M}$ is a topological ball for all $0<\eta < r_c-|c-y|$, where $r_c$ is the second critical value of the distance function to *c* restricted to $\mathcal {M}$. In other words $r_c$ is the radius for which the ball centred on *c* is tangent to $\mathcal {M}$ for the second time.

Let us now assume that there exists a point $z\ne y$ of $\mathcal {M}$ such that $r_c=| c-z| > | c-y|$ where the ball $B(c,r_c)$ is tangent to $\mathcal {M}$. Corollary 2 and the assumption that the radius of *B* is strictly less than the reach now gives that $B(c,r_c)$ contains no points of $\mathcal {M}$ in its interior. This cannot be, because *y* lies inside this ball, meaning no such point *z* can exist. $\square$

### Bounds for $C^{1,1}$ submanifolds

We shall now give a bound on the angle between sufficiently close tangent spaces based on elementary arguments. Here we use elementary methods in the sense that we do not rely on differential geometry, although we will use the topological ball result. The bound we find here also encompasses the $C^{1,1}$ case, and thus holds for arbitrary manifolds of positive reach.

#### From manifold to tangent space and back

We start with the following lemma, which is due to Federer. It bounds the distance of a point $q\in \mathcal {M}$ to the tangent space $T_p\mathcal {M}$ at a nearby point $p \in \mathcal {M}$. We include a proof for completeness.

##### Lemma 10

(Distance to tangent space, Theorem 4.8(7) of Federer (1959)) Let $p,q \in \mathcal {M}\subset \mathbb {R}^d$ such that $|p-q| < \mathrm {rch}(M)$. We have

8 $$\begin{aligned} \sin \angle ([pq], T_p \mathcal {M}) \le \frac{|p-q|}{2\, \mathrm {rch}(\mathcal {M})}, \end{aligned}$$

and

9 $$\begin{aligned} d_{\mathbb {E} } (q , T_p \mathcal {M}) \le \frac{|p-q|^2}{2\, \mathrm {rch}(\mathcal {M})}. \end{aligned}$$

##### Proof

Write $v= \pi _{T_p\mathcal {M}} (q)$. Consider the plane *H* in which *v*, *q* and *p* lie. Let in addition $B_1$, $B_2$ be the two disks in *H* that are tangent to $\mathcal {M}$ at *p* and thus to $T_p\mathcal {M}$ with radius $\mathrm {rch}(\mathcal {M})$. Due to Lemma 2*q* cannot lie inside the interior of $B_1$ nor $B_2$. Let us now extend the line [*vq*] and call the first intersection of this line with $B_1$, $q_1$ and with $B_2$, $q_2$. We call the centres of $B_1$ and $B_2$, $c_1$ and $c_2$, and the angles $\angle ([q_1 c_1],[c_1 p]) =\angle ([q_2 c_2],[c_2 p]) =\theta$. We find that $|v-p|= \mathrm {rch}(\mathcal {M}) \sin \theta$, while

$$\begin{aligned} |q-v| \le |v-q_1| = |v-q_2| = (1 -\cos \theta ) \mathrm {rch}(\mathcal {M}) . \end{aligned}$$

This gives us

$$\begin{aligned} |q-v| \le \left( 1- \sqrt{1- \left( \frac{|v-p|}{\mathrm {rch}(\mathcal {M}) } \right) ^2} \right) \mathrm {rch}(\mathcal {M}) \le \frac{1}{2} \frac{|v-p|^2}{ \mathrm {rch}(\mathcal {M})}+ \frac{1}{2} \frac{|v-p|^4}{ \mathrm {rch}(\mathcal {M})^3}, \end{aligned}$$

using Taylor’s theorem. $\square$

Next lemma establishes the converse statement of the distance bounds in Lemma 10. It is an improved version of Lemma B.2 in Boissonnat et al. (2013). This result too can be traced back to Federer (1959), in a slightly different guise. Before we give the lemma we first introduce the following notation.

##### Definition 2

Let $C(T_p \mathcal {M},r_1, \tilde{r} )$ denote the ‘filled cylinder’ given by all points that project orthogonally onto a ball of radius $r_1$ in $T_p \mathcal {M}$ and whose distance to this ball is less than $\tilde{r}$.

In the following lemma we prove for all points $v \in T_p\mathcal {M}$, such that $|v-p|$ is not too large, that a pre-image on $\mathcal {M}$, if it exists, under the projection to $T_p\mathcal {M}$ cannot be too far from $T_p\mathcal {M}$. The existence of such a point on $\mathcal {M}$ is proven below.

##### Lemma 11

(Distance to Manifold) Suppose that $v \in T_p \mathcal {M}$ and $|v-p| < \mathrm {rch}(\mathcal {M})$. Let $q= \pi _{(\mathcal {M}\rightarrow T_p \mathcal {M}) } ^{-1} (v)$ be the inverse of the (restricted) projection $\pi _{T_p \mathcal {M}}$ from $\mathcal {M}\cap C(T_p \mathcal {M},\mathrm {rch}(\mathcal {M}),\mathrm {rch}(\mathcal {M}))$ to $T_p\mathcal {M}$ of *v*, if it exists. Then

$$\begin{aligned} | q-v|\le \left( 1- \sqrt{1- \left( \frac{|v-p|}{\mathrm {rch}(\mathcal {M}) } \right) ^2} \right) \mathrm {rch}(\mathcal {M}) \le \frac{1}{2} \frac{|v-p|^2}{ \mathrm {rch}(\mathcal {M})}+ \frac{1}{2} \frac{|v-p|^4}{ \mathrm {rch}(\mathcal {M})^3}. \end{aligned}$$

##### Remark 1

**Fig. 4:**
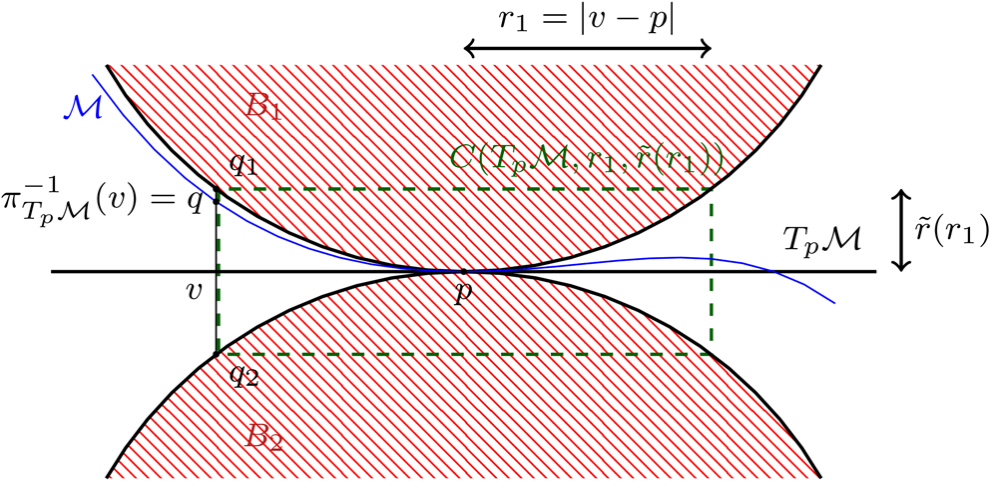
The set of all tangent balls to the tangent space of radius $\mathrm {rch}(\mathcal {M})$ bounds the region in which $\mathcal {M}$ can lie. Here we depict the 2 dimensional analogue

It follows immediately that $\mathcal {M}\cap C(T_p \mathcal {M},r_1,\mathrm {rch}(\mathcal {M}))\subset C(T_p \mathcal {M},r_1, \tilde{r}(r_1) )$, with

10 $$\begin{aligned} \tilde{r}(r_1) =\left( 1- \sqrt{1- \left( \frac{r_1}{\mathrm {rch}(\mathcal {M}) } \right) ^2} \right) \mathrm {rch}(\mathcal {M}), \end{aligned}$$

for any $r_1 \le \mathrm {rch}(\mathcal {M})$. This cylinder is indicated in green in Fig. 4. Let $C_{\text {top/bottom}} (T_p \mathcal {M},r_1,\tilde{r}(r_1))$ denote the subset of $C (T_p \mathcal {M},r_1,\tilde{r}(r_1))$ that projects orthogonally onto the open ball of radius $r_1$ in $T_p\mathcal {M}$ and lies a distance $\tilde{r}(r_1)$ away. We also see that $\mathcal {M}\cap C_{\text {top/bottom}}(T_p \mathcal {M},r_1,\tilde{r}(r_1) ) =\emptyset$ and that $\mathcal {M}\cap C(T_p \mathcal {M},r_1,\mathrm {rch}(\mathcal {M})) \cap N_p\mathcal {M}=\{p\}$. We write

$$\begin{aligned} C_{\text {side rim}} (T_p \mathcal {M},r_1,\tilde{r}(r_1)) =\partial C (T_p \mathcal {M},r_1,\tilde{r}(r_1)) {\setminus } C_{\text {top/bottom}} (T_p \mathcal {M},r_1,\tilde{r}(r_1)). \end{aligned}$$

**Fig. 5:**
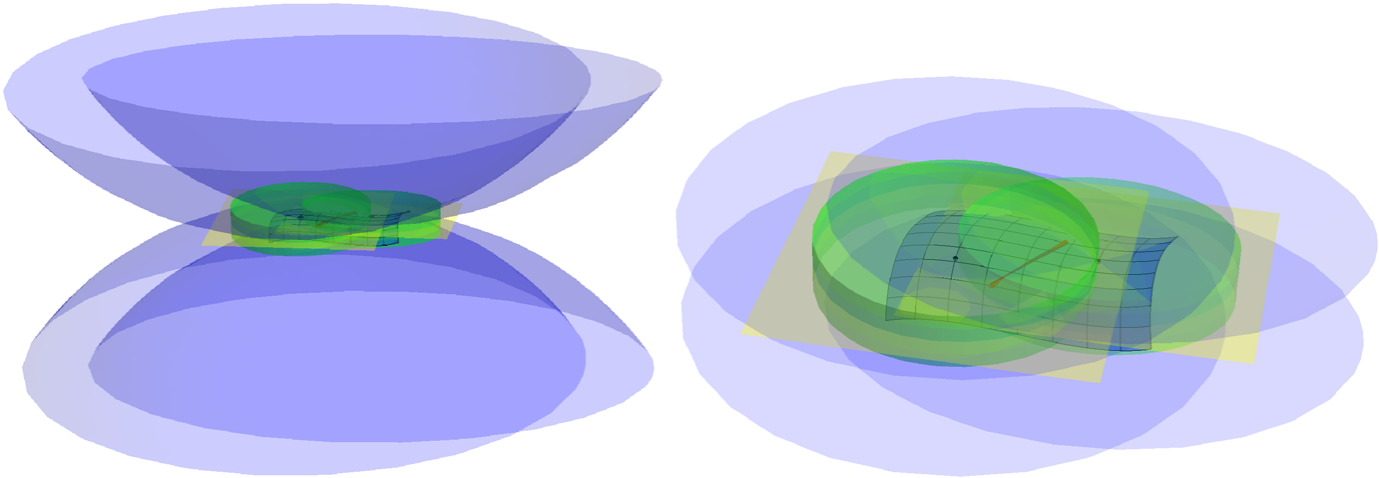
The tangent spaces $T_p \mathcal {M}$ and $T_q \mathcal {M}$ are drawn in yellow. The cylinders $C(T_p \mathcal {M},r_1, \tilde{r})$ and $C(T_q \mathcal {M},r_1, \tilde{r})$ are indicated in green. The red line segment lies in both cylinders and therefore its angle with both $T_p \mathcal {M}$ and $T_q \mathcal {M}$ is small (color figure online)

#### The angle bound

This section revolves around the following observation: If $r_1$ roughly the distance between *p* and *q*, there is a significant part of $\mathcal {M}$ that is contained in the intersection $C(T_p \mathcal {M},r_1, \tilde{r}) \cap C(T_q \mathcal {M},r_1, \tilde{r})$, where we abbreviated $\tilde{r} (r_1)$ to $\tilde{r}$. In particular any line segment, whose length is denoted by $\ell$, connecting two points in $\mathcal {M}\cap C(T_p \mathcal {M},r_1, \tilde{r}) \cap C(T_q \mathcal {M},r_1, \tilde{r})$ is contained in both $C(T_p \mathcal {M},r_1, \tilde{r})$ and $C(T_q \mathcal {M},r_1, \tilde{r})$. If this line segment is long, the angle with both $T_p \mathcal {M}$ and $T_q \mathcal {M}$ is small. This bounds the angle between $T_p \mathcal {M}$ and $T_q \mathcal {M}$, see Fig. 5.

We start with the following simple observation:

##### Remark 2

**Fig. 6:**
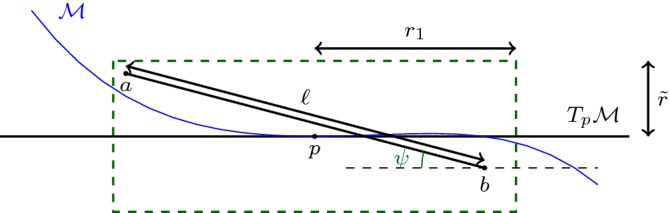
An illustration of the notation used in Remark 2

Let [*ab*] be a line segment with length $\ell$ that is contained in $C(T_p \mathcal {M},r_1,\tilde{r} )$. Then the angle $\psi$ between [*ab*] and $T_p \mathcal {M}$ is bounded by $\sin (\psi ) \le \frac{2 \tilde{r}}{\ell }$ (Fig. 6).

We now need the following corollary of Proposition 1:

##### Corollary 4

We have:

1. For any ball *B*(*p*, *r*) of radius $r< \mathrm {rch}(\mathcal {M})$ centred at $p \in \mathcal {M}$, $B(p,r) \cap \mathcal {M}$ is a topological ball.
2. For every $0<r< \mathrm {rch}(\mathcal {M})$, $\partial (B(p,r) \cap \mathcal {M})$ is contained in a set homeomorphic to $C_{\text {side rim}} (T_p \mathcal {M},r,\tilde{r}(r))$, this homeomorphism is a projection, which is denoted by $h_r$ and indicated in Fig. 8 in green.
3. There exists an isotopy from the image of $\partial (B (p,r) \cap \mathcal {M})$ under the homeomorphism from $C_{\text {side rim}} (T_p \mathcal {M},r,\tilde{r}(r))$ to the sphere that is the boundary of the open ball of radius *r* in $T_p\mathcal {M}$.

##### Proof

The first observation is a straightforward consequence of Proposition 1 and the definition of the reach.

We have that $B(p,r) \subset C(T_p \mathcal {M},r,\mathrm {rch}(\mathcal {M}))$, so thanks to Remark 1 we see that

$$\begin{aligned} B(p,r) \cap \mathcal {M}\cap N_p\mathcal {M}= \mathcal {M}\cap C(T_p \mathcal {M},r_1,\mathrm {rch}(\mathcal {M})) \cap N_p\mathcal {M}=\{p\}. \end{aligned}$$

Because $\mathcal {M}$ does not have a boundary, we see that $\partial (B(p,r)\cap \mathcal {M}) \subset \partial (B(p,r)) {\setminus } N_p\mathcal {M}$. The set $\partial (B(p,r)) {\setminus } N_p\mathcal {M}$ is a sphere with a $d-n$ dimensional linear space removed and thus homeomorphic to the open cylinder ($\mathbb {S}^{n}\times (-1,1)^{d-n}$). This means its closure is a closed cylinder and thus homeomorphic to $C_{\text {side rim}} (T_p \mathcal {M},r,\tilde{r}(r))$. This gives us the second observation.

The third observation is obviously true for sufficiently small $r=\epsilon$, because the tangent space is the first order approximation of the manifold. Because the second observation holds for any $r' \le \mathrm {rch}(\mathcal {M})$, the third observation follows. Roughly speaking, the isotopy can be found by following $\partial (B(p,r')\cap \mathcal {M})$ from $r' =r$ to the limit of $r'$ going to zero.

**Fig. 7:**
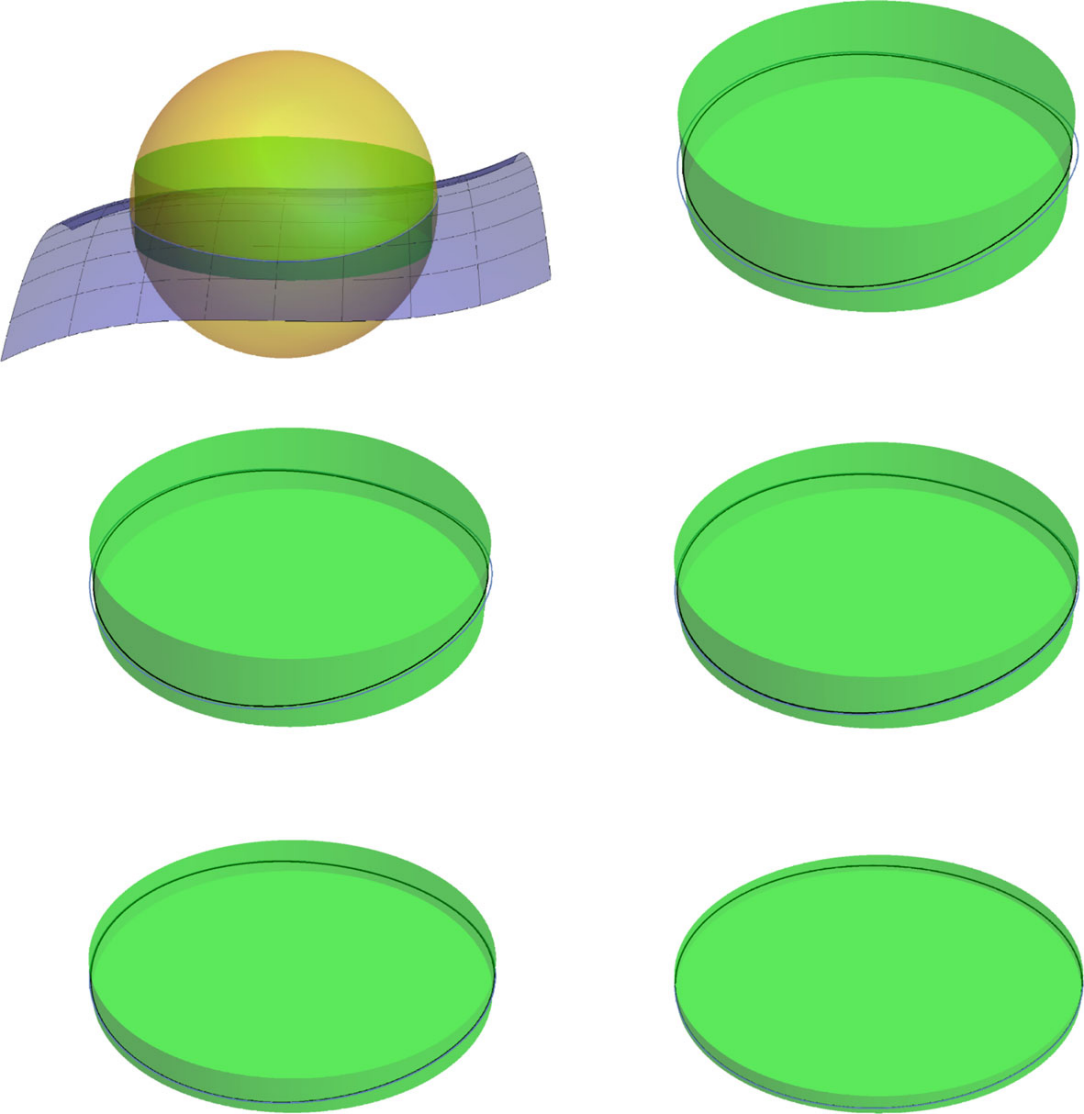
Overview of the proof of the third point of Corollary 4. In the first image we see the intersection between the sphere $\partial B(p,r)$ and the manifold $\mathcal {M}$. The following figures focus on the intersection $\partial B(p,r)\cap \mathcal {M}$ in blue and its projection in black. This is depicted for smaller and smaller radii of the sphere, but rescaled to the size of the fist image. Notice that the curve of intersection tends to the circle (color figure online)

More precisely the isotopy can be understood as follows, see also Fig. 7:

- Thanks to Proposition 1, $\mathcal {M}\cap \partial B(p,r)$ is a topological sphere. For each $0<r'< \mathrm {rch}(\mathcal {M})$, $h_{r'}(\partial (B(p,r') \cap M))$ lies on $C_{\text {side rim}} (T_p \mathcal {M},r',\tilde{r}(r'))$.
- In turn $C_{\text {side rim}} (T_p \mathcal {M},r',\tilde{r}(r'))$ can be rescaled in the radial direction such that the image is contained in $C_{\text {side rim}} (T_p \mathcal {M},r,\tilde{r}(r))$. This rescaling is denoted by the map $R_{r'\rightarrow r}$.
- The map $R_{r'\rightarrow r}(h_{r'}(\partial (B(p,r') \cap M)))$ now gives the isotopy, because the limit $\lim _{r' \rightarrow 0 } R_{r'\rightarrow r}(h_{r'}(\partial (B(p,r') \cap M)))$ is in fact the sphere in the tangent space.

$$\square $$

For the existence of the line segment that is contained in both $C(T_p \mathcal {M},r_1, \tilde{r})$ and $C(T_q \mathcal {M},r_1, \tilde{r})$ we need the following corollary of Proposition 1:

##### Corollary 5

For each $v \in T_p \mathcal {M}$ such that $|v-p| <\frac{\sqrt{3}}{2} \mathrm {rch}(\mathcal {M})$ there exists at least one point $\pi _{T_p \mathcal {M}}^{-1}(v)$.

**Fig. 8:**
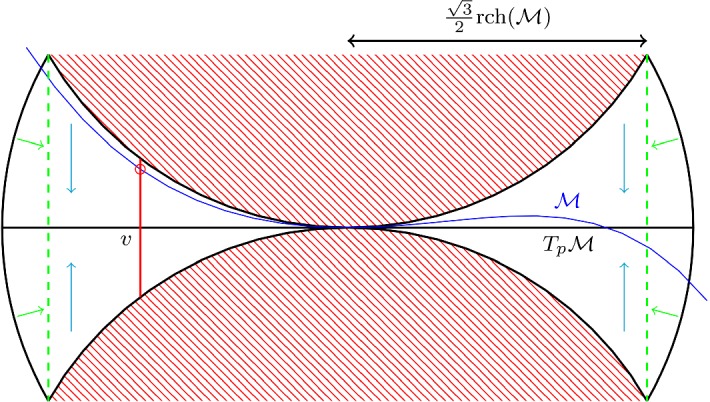
The manifold $\mathcal {M}$ in a neighbourhood of the point *p* lies in region bounded by all tangent balls of $T_p \mathcal {M}$ at *p*, indicated by the red balls. The projection on the boundary of $C(T_p \mathcal {M}, \frac{\sqrt{3}}{2} \mathrm {rch}(\mathcal {M}) , \frac{1}{2} \mathrm {rch}(\mathcal {M}) )$ is indicated in green. The projection onto the tangent page is indicated in cyan (color figure online)

##### Proof

The proof, by contradiction, is completely pictorial in nature, see Fig. 8. So let us suppose that there exists a $v \in T_p \mathcal {M}$ with $|v-p| <\frac{\sqrt{3}}{2} \mathrm {rch}(\mathcal {M})$ such that there does not exist a $\pi _{T_p \mathcal {M}}^{-1}(v)$. Consider the ball $B(p, \mathrm {rch}(\mathcal {M}) )$. $\mathcal {M}\cap B(p, \mathrm {rch}(\mathcal {M}) )$ is a topological ball, by Corollary 4. We now map (radially) the part of this ball outside the cylinder $C(T_p \mathcal {M}, \frac{\sqrt{3}}{2} \mathrm {rch}(\mathcal {M}) , \frac{1}{2} \mathrm {rch}(\mathcal {M}) )$ onto the boundary of $C(T_p \mathcal {M}, \frac{\sqrt{3}}{2} \mathrm {rch}(\mathcal {M}) , \frac{1}{2} \mathrm {rch}(\mathcal {M}) )$, as indicated in Fig. 8. We then project everything onto $T_p \mathcal {M}$. By Corollary 4 one has that the result is the image of a topological ball whose boundary coincides with the boundary of $B_{T_p \mathcal {M}} ( \frac{\sqrt{3}}{2} \mathrm {rch}(\mathcal {M}))$. However because we assumed that there did not exist a $\pi _{T_p \mathcal {M}}^{-1}(v)$, this image of the topological ball is topologically non-trivial, which yields a clear contradiction, because if there is a puncture the boundary of the topological ball would no longer be homologically trivial. $\square$

##### Theorem 3

Let $|p-q|\le \mathrm {rch}(\mathcal {M})/3$, then the angle $\varphi$ between $T_p\mathcal {M}$ and $T_q \mathcal {M}$ is bounded by

$$\begin{aligned} \sin \frac{ \varphi }{2}&\le \frac{ \left( 1- \sqrt{1- \alpha ^2} \right) }{\sqrt{ \frac{\alpha ^2}{4} - \left( \frac{\alpha ^2}{2 }+ 1- \sqrt{1- \alpha ^2} \right) ^2 } } \\&\simeq \alpha + 9 \alpha ^3/4, \end{aligned}$$

where $$\alpha = |p-q|/\mathrm {rch}(\mathcal {M})$$.

##### Proof

The idea of the proof is pictorial, as we have seen in the overview in Fig. 5 and below. We shall now give the details.

**Fig. 9:**
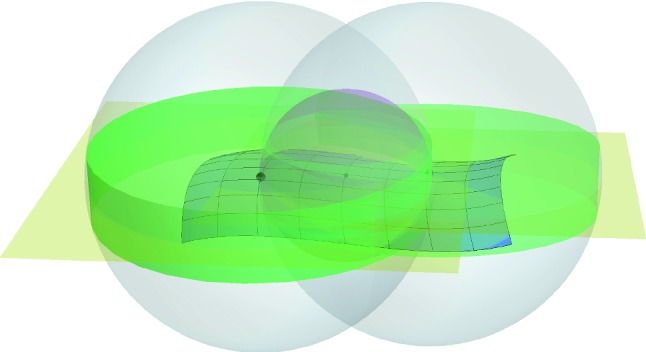
The ball $B(m,\frac{|p-q|}{2})$ lies in both $B(p,|p-q|)$ and $B(q,|p-q|)$

We consider the balls of radius $|p-q|$ centred at *p* and *q* respectively. The ball of radius $\frac{|p-q|}{2}$ centred at the midpoint $m= \frac{p+q}{2}$ is clearly contained in both larger balls, being $B(p,|p-q|)$ and $B(q,|p-q|)$, as indicated in Fig. 9.

**Fig. 10:**
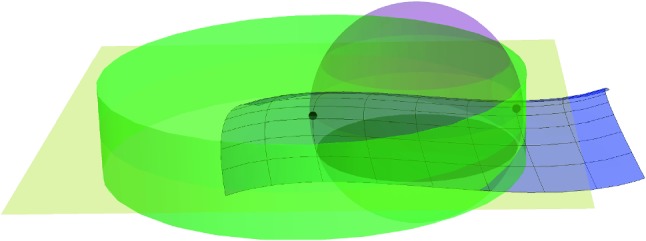
$B_{T_p\mathcal {M}}(\ell )$ is the dark disk that lies in the sphere

We now note that $\mathcal {M}\cap B(m, \frac{|p-q|}{2} )$ is contained in both the cylinders $C(T_p \mathcal {M},|p-q|, \tilde{r}(|p-q|) )$ and $C(T_q \mathcal {M},|p-q|, \tilde{r}(|p-q|) )$. Moreover, there exists an *n*-dimensional ball $B_{T_p\mathcal {M}}(\ell )$ of diameter $\ell$ in $T_p\mathcal {M}$ (the dark disk in Fig. 10) such that $\pi _{T_p\mathcal {M}}^{-1}(x) \in B(m, \frac{|p-q|}{2} )$ for all $x \in B_{T_p\mathcal {M}}(\ell )$. Determining $\ell$ is the only part of this proof for which we have to do some calculations, which we postpone until the end of the proof.

**Fig. 11:**
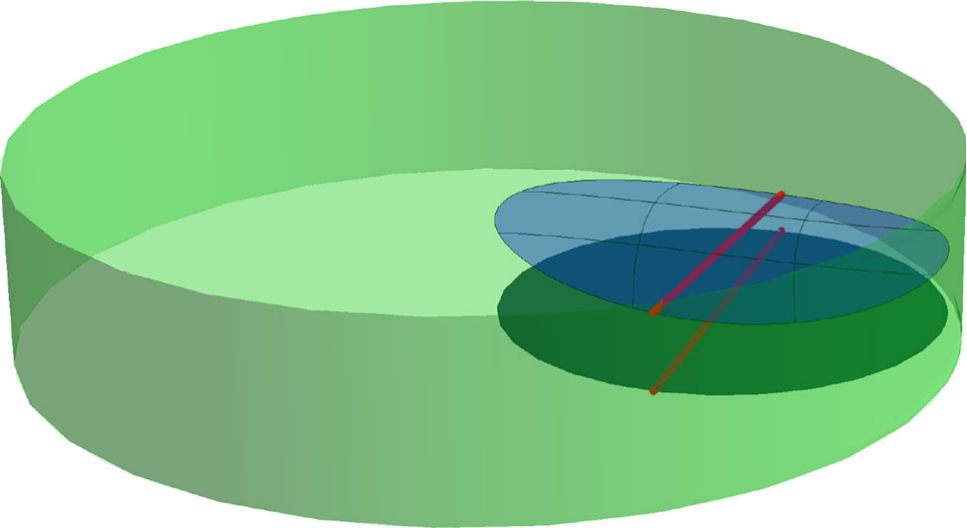
The line segment connecting two antipodal point $y_1, y_2$ on the sphere $\partial B_{T_p\mathcal {M}}(\ell )$ is indicated as a dotted red line and the line segment connecting $\pi _{T_p\mathcal {M}}^{-1}(y_1)$ and $\pi _{T_p\mathcal {M}}^{-1}(y_2)$ is indicated in red (color figure online)

For each direction in $T_p\mathcal {M}$ we can consider the line segment connecting two antipodal point $y_1, y_2$ on the sphere $\partial B_{T_p\mathcal {M}}(\ell )$ and the line segment connecting $\pi _{T_p\mathcal {M}}^{-1}(y_1)$ and $\pi _{T_p\mathcal {M}}^{-1}(y_2)$, see Fig. 11. These two points exist because of Corollary 5. This line segment has at least length $\ell$. Moreover it lies in both $C(T_p \mathcal {M},|p-q|, \tilde{r}(|p-q|) )$, $C(T_q \mathcal {M},|p-q|, \tilde{r}(|p-q|) )$, with $\tilde{r}$ as in (10).

We now have a line segment for each direction in $T_p \mathcal {M}$ that is close to that direction in $T_p \mathcal {M}$, because it lies in $C(T_p \mathcal {M},|p-q|, \tilde{r}(|p-q|) )$, and is close to $T_q\mathcal {M}$, because the line segment lies in $C(T_q \mathcal {M},|p-q|, \tilde{r}(|p-q|) )$. If this line segment is not too short compared to $\tilde{r}(|p-q|)$, Lemmas 10, 11 and Remark 2 give us that the angle between $T_p \mathcal {M}$ and $T_q \mathcal {M}$ is small.

**Fig. 12:**
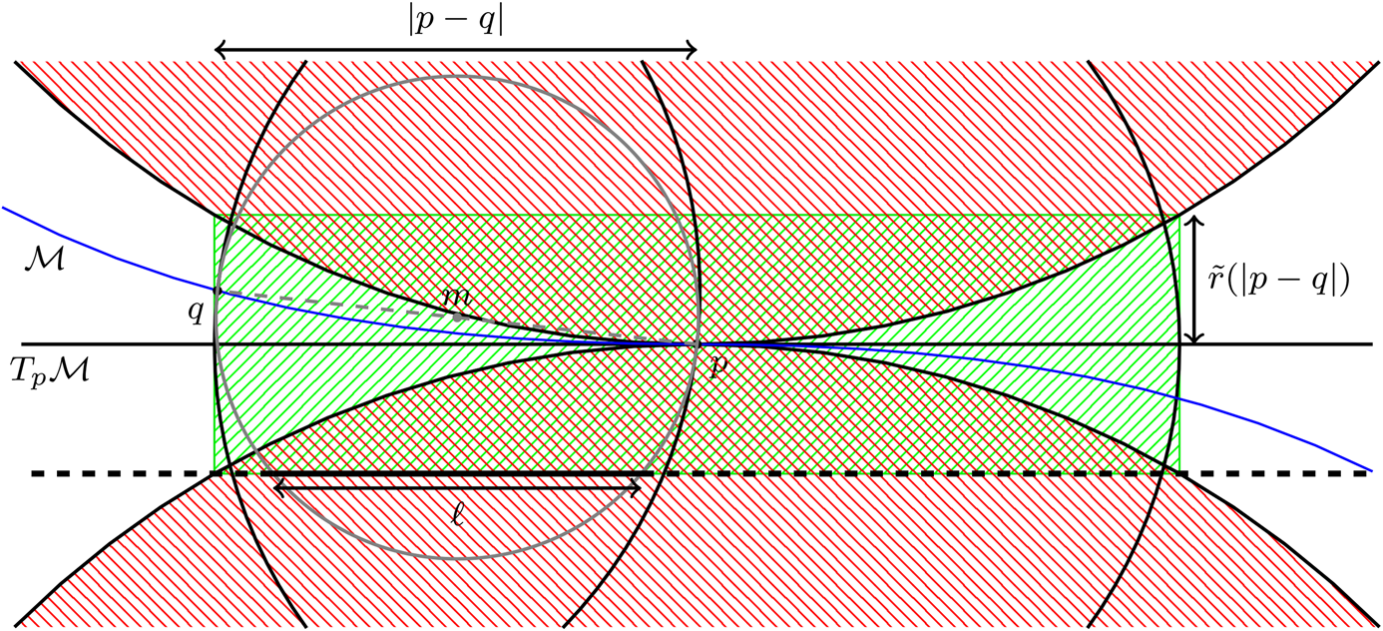
The intersection region of the balls centred at *p* and *q* with radius $|p-q|$

The only thing which is left is to give a lower bound $\ell$. For this we shall use Fig. 12. We shall denote the orthogonal translation of $T_p\mathcal {M}$ that goes through a point *x* by $\text {Trans}_x (T_p\mathcal {M})$. Let $\text {Trans}_\text {max} (T_p\mathcal {M})$ be the orthogonal translation of $T_p\mathcal {M}$ to the furthest possible affine subspace from *q*, such that the intersection of $\text {Trans} (T_p\mathcal {M})$ and $C(T_p \mathcal {M},|p-q|, \tilde{r}(|p-q|) )$ is nonempty. $\text {Trans}_\text {max} (T_p\mathcal {M})$ is indicated by a thick dashed line in Fig. 12. The radius of the intersection of $\text {Trans}_\text {max} (T_p\mathcal {M})$ with $B(m,\frac{|p-q|}{2})$ gives us $\ell /2$.

Because Lemma 10 gives us that *m* lies at most $\frac{|p-q|^2}{2 \mathrm {rch}(\mathcal {M})}$ from $T_p\mathcal {M}$ and the distance between $\text {Trans}_\text {max} (T_p\mathcal {M})$ and $T_p\mathcal {M}$ is $\tilde{r}(|p-q|)$ we have, by Pythagoras,

$$\begin{aligned} (\ell /2)^2 = \left( \frac{|p-q|}{2}\right) ^2 - \left( \frac{|p-q|^2}{2 \mathrm {rch}(\mathcal {M})}+ \tilde{r}(|p-q|) \right) ^2. \end{aligned}$$

Using Remark 2, we see that

$$\begin{aligned} \sin \frac{ \varphi }{2} \le \frac{2 \tilde{r}(|p-q|)}{\ell },\ \end{aligned}$$

where the factor 2 on the left hand side is due to the fact that we apply the bound twice, once for each cylinder. To be precise we have used

$$\begin{aligned} \phi = \angle (T_p\mathcal {M},T_q \mathcal {M}) \le&\sup _{y_1,y_2 \in \partial B_{T_p\mathcal {M}}(\ell )} \angle (T_p \mathcal {M},\pi _{T_p\mathcal {M}}^{-1}(y_1)-\pi _{T_p\mathcal {M}}^{-1}(y_2))\\&+ \angle (T_q \mathcal {M},\pi _{T_p\mathcal {M}}^{-1}(y_1)-\pi _{T_p\mathcal {M}}^{-1}(y_2)), \end{aligned}$$

where we understand that the supremum is taken over antipodal points $y_1$ and $y_2$ in $\partial B_{T_p\mathcal {M}}(\ell )$ and $\sin ( a+b) \le \sin (a)+\sin (b)$.

Combining the results yields

$$\begin{aligned} \sin \frac{ \varphi }{2}&\le \frac{ \tilde{r}(|p-q|)}{\sqrt{\left( \frac{|p-q|}{2}\right) ^2 - \left( \frac{|p-q|^2}{2 \mathrm {rch}(\mathcal {M})}+ \tilde{r}(|p-q|) \right) ^2 } }, \\&= \frac{ \left( 1- \sqrt{1- \left( \frac{|p-q|}{\mathrm {rch}(\mathcal {M}) } \right) ^2} \right) \mathrm {rch}(\mathcal {M}) }{\sqrt{\left( \frac{|p-q|}{2}\right) ^2 - \left( \frac{|p-q|^2}{2 \mathrm {rch}(\mathcal {M})}+ \left( 1- \sqrt{1- \left( \frac{|p-q|}{\mathrm {rch}(\mathcal {M}) } \right) ^2} \right) \mathrm {rch}(\mathcal {M}) \right) ^2 } } \\&= \frac{ \left( 1- \sqrt{1- \alpha ^2} \right) }{\sqrt{ \frac{\alpha ^2}{4} - ( \frac{\alpha ^2}{2 }+ 1- \sqrt{1- \alpha ^2} )^2 } } \\&\simeq \alpha + 9 \alpha ^3/4, \end{aligned}$$

where $$\alpha = |p-q|/\mathrm {rch}(\mathcal {M})$$. $$\square $$

##### Remark 3

The bound we presented above can be tightened by further geometric analysis, in particular by splitting $T_p\mathcal {M}$ into the span of $\pi _{T_p \mathcal {M}} (q)-p$ and its orthocomplement. However we chose to preserve the elementary character of the argument. At the moment the bound is about half as good for small $\alpha$ as the smooth bound. The bound on $\alpha$ itself is a third of what one can prove in the smooth setting. It is not clear that this gap can be completely closed with these techniques.

With the bound on the angles between the tangent spaces it is not difficult to prove that the projection map is locally a diffeomorphism, as has been done in Niyogi et al. (2008).

## Metric distortion and geodesic convexity for $\mathbf {C^2}$ submanifolds

In this section we prove the results on distortion and geodesic convexity for $C^2$ manifolds. The first part of this exposition is the result of straightforwardly combining the work of Niyogi et al. (2008), and the two dimensional analysis of Attali et al. (2007) with some observations concerning the reach. The proof of geodesic convexity of the intersection of the manifold and a sufficiently small ball (Corollary 1 for $C^{1,1}$ manifolds and Theorem 4 for $C^2$ manifolds) uses the same techniques as those we have seen in Sect. 3, and are again based on the simple observation made in Lemma 4. We have included the smooth analysis in the final section because we feel that it gives a different perspective on the problem. Some of the intermediate results, in this smooth setting, may also be of independent interest.

### Metric distortion

We remind ourselves that Lemma 5 says that if $\gamma (t)$ is a geodesic parametrized according to arc length whose length $\ell$ therefore equals $d_\mathcal {M}(p,q)$, such that $\gamma (0)=p$ and $\gamma (\ell )=q$, we have that,

7 $$\begin{aligned} \angle \dot{\gamma }(a) \dot{\gamma }(b) \le \frac{|a-b| }{\mathrm {rch}(\mathcal {M})} . \end{aligned}$$

We now have the following, which is the straightforward generalization of Property I of Attali et al. (2007) to arbitrary dimension and using the reach:

#### Lemma 12

Let $p,q \in \mathcal {M}$ be such that $d_\mathcal {M}(p,q) \le \pi \mathrm {rch}(\mathcal {M})$, then

$$\begin{aligned} \sin \left( \frac{ d_\mathcal {M}(p,q)}{2\mathrm {rch}(\mathcal {M})} \right) \le \frac{|p-q|}{2 \, \mathrm {rch}(\mathcal {M})}. \end{aligned}$$

#### Proof

The length of $\gamma$ in the direction $\dot{\gamma }(\frac{\ell }{2})$ is

$$\begin{aligned} \left\langle q-p , \dot{\gamma } \left( \frac{\ell }{2} \right) \right\rangle&= \int _{0}^{\ell } \langle \dot{\gamma }(s), \dot{\gamma }(\ell /2) \rangle \mathrm {d}s \\&= \int _{0}^{\ell /2} \langle \dot{\gamma }(s), \dot{\gamma }(\ell /2) \rangle \mathrm {d}s +\int _{\ell /2}^{\ell } \langle \dot{\gamma }(s), \dot{\gamma }(\ell /2) \rangle \mathrm {d}s \\&\ge \int _{0}^{\ell /2} \cos \frac{|s-\ell /2| }{\mathrm {rch}(\mathcal {M})} \mathrm {d}s +\int _{\ell /2}^{\ell } \cos \frac{|s-\ell /2| }{\mathrm {rch}(\mathcal {M})} \mathrm {d}s \\&= 2 \, \mathrm {rch}(\mathcal {M}) \sin \left( \frac{ \ell }{2\mathrm {rch}(\mathcal {M})} \right) . \end{aligned}$$

Because $|q-p| \ge \langle q-p , \dot{\gamma }(\frac{\ell }{2}) \rangle$, the result follows. $\square$

This bound is tight as it is attained on the sphere of the appropriate dimension.

### Convexity

We now prove that the intersection of a ball with radius less than the reach with the manifold is geodesically convex, using differential geometric techniques. To prove this we first give a bound on the distance between (a sufficiently short) geodesic on the manifold and the straight line segment connecting the endpoints of the geodesic. In fact we’ll see that the worst case scenario is the sphere with radius reach. Secondly we’ll show that if points are closer than $2 \mathrm {rch}(\mathcal {M})$ in the ambient space, they are also close on the manifold. The main result is a fairly straightforward consequence of these two lemmas.

Here we shall use the estimate (7) to prove the following:

#### Lemma 13

Let $p,q \in \mathcal {M}$ be such that $d_\mathcal {M}(p,q) <\pi \mathrm {rch}(\mathcal {M})$ and let $\gamma (t)$ be a minimizing geodesic parametrized by arc length connecting *p* and *q* with length $\ell$, then

$$\begin{aligned} d_\mathbb {E}(\gamma (t) , [pq]) \le \mathrm {rch}(\mathcal {M}) \left( \cos \frac{|\ell /2-t| }{\mathrm {rch}(\mathcal {M})} -\cos \frac{\ell }{2 \mathrm {rch}(\mathcal {M})}\right) , \end{aligned}$$

for $$0\le t \le \ell $$.

#### Proof

**Fig. 13:**
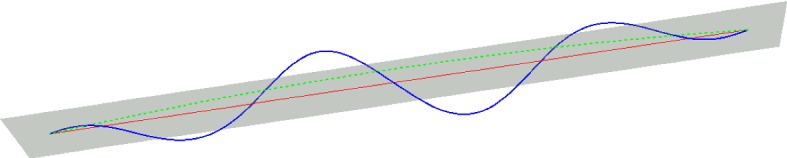
A sketch of the curves $\gamma (t)$ (blue), $\tilde{\gamma }(t)$ (green), and the line segment [*pq*] (red). The $\rho ,z$-plane is indicated in greyish green, the ‘*z*’-direction is the direction of the segment [*pq*] (color figure online)

We shall denote the orthogonal projection onto [*pq*] by $\pi _{[pq]}$ and the direction of the line segment [*pq*] by *z*. We now consider the two dimensional curve $\tilde{\gamma }(t)= (\tilde{\gamma }_z(t),\tilde{\gamma }_\rho (t))= (\pi _{[pq]}(\gamma (t)), |\gamma (t)-\pi _{[pq]}(\gamma (t) )| )$. The geometric interpretation is the following: We first consider $\gamma (t)$ in cylindrical coordinates, where we regard the line that extends [*pq*] as the ‘*z*-axis’. We then project on the radial $\rho$ and ‘*z*’-direction. We also refer to the unit vector in the radial direction as $\rho$ (Fig. 13).

Observe that $\dot{\tilde{\gamma }}(t)$ is the projection on the $\rho ,z$-plane of $\dot{\gamma }(t)$ and thus

$$\begin{aligned} \angle (\dot{\tilde{\gamma }}(a) ,\dot{\tilde{\gamma }}(b) )\le \angle ( \dot{\gamma }(a), \dot{\gamma }(b)) , \end{aligned}$$

because any projection decreases angles. Using (7) we now see that

11 $$\begin{aligned} \angle (\dot{\tilde{\gamma }}(a), \dot{\tilde{\gamma }}(b)) \le \frac{|a-b| }{\mathrm {rch}(\mathcal {M})}. \end{aligned}$$

We also note that $|\dot{\tilde{\gamma }}(t)|\le 1$.

Let $s^*\in [0 , \ell ]$ be a point such that $\frac{d}{dt} (\tilde{\gamma }_\rho (t)) \mid _{t=s^*} = 0$, that is $\dot{\tilde{\gamma }}(s^*)$ lies in the *z*-direction. There exists such an $s^*$ for there is a point where the maximum of $\tilde{\gamma }_\rho (t)$ is attained. By possibly interchanging the roles of *p* and *q* we can assume that $s^*\le \ell /2$.

We now have the following estimate

$$\begin{aligned} d_{\mathbb {E}}(\gamma (s^*) , [pq])&\le \left| \int _{0}^{s^*} \langle \rho , \dot{\tilde{\gamma }}(s) \rangle \mathrm {d}s \right| \\&\le \int _{0}^{s^*} |\langle \rho , \dot{\tilde{\gamma }}(s) \rangle | \mathrm {d}s \\&\le \int _{0}^{s^*} \cos \angle ( \rho ,\dot{\tilde{\gamma }}(s) )\mathrm {d}s \\&= \int _{0}^{s^*} \sin \angle ( z, \dot{\tilde{\gamma }}(s)) \mathrm {d}s \\&= \int _{0}^{s^*} \sin \angle ( \dot{\tilde{\gamma }}(s^*), \dot{\tilde{\gamma }}(s) ) \mathrm {d}s \\&\le \int _{0}^{s^*} \sin \frac{|s-s^*| }{\mathrm {rch}(\mathcal {M})} \mathrm {d}s \\&= \mathrm {rch}(\mathcal {M}) \left( 1-\cos \frac{s^* }{\mathrm {rch}(\mathcal {M})} \right) , \end{aligned}$$

where the third inequality is due to the fact that $|\dot{\gamma }|\le 1$ and the last inequality is due to (11). It is clear that the bound is maximized if $s^*=\ell /2$. This maximum is attained for the sphere of the appropriate dimension.

We can now do the same analysis for any $t \in [0 ,s^*]$. We see that

$$\begin{aligned} d_{\mathbb {E}}(\gamma (t) , [pq])&\le \left| \int _{0}^{t } \langle \rho , \dot{\tilde{\gamma }}(s) \rangle \mathrm {d}s \right| \\&\le \int _{0}^{t} \sin \angle ( \dot{\tilde{\gamma }}(s^*) ,\dot{\tilde{\gamma }}(s) ) \mathrm {d}s \\&\le \int _{0}^{t} \sin \frac{|s-s^*| }{\mathrm {rch}(\mathcal {M})} \mathrm {d}s \\&\le \mathrm {rch}(\mathcal {M}) \left( \cos \frac{|s^*-t| }{\mathrm {rch}(\mathcal {M})} -\cos \frac{s^* }{\mathrm {rch}(\mathcal {M})} \right) , \end{aligned}$$

which again is maximized if $s^*=\ell /2$ and attained for the sphere. $\square$

We also need the following lemma:

#### Lemma 14

Let $\mathcal {M}$ be a compact $C^2$ manifold and $p,q \in \mathcal {M}$ be such that $|p-q| < 2 \mathrm {rch}(\mathcal {M})$, then $d_\mathcal {M}(p,q) < \pi \mathrm {rch}(\mathcal {M})$.

#### Proof

**Fig. 14:**
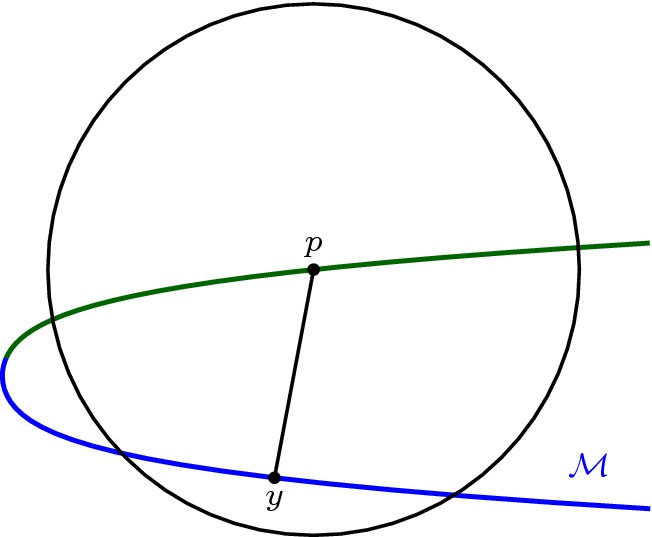
The set $B_{\mathcal {M}}(p,\pi \mathrm {rch}(\mathcal {M}))$ is indicated is green, while $\mathcal {M}{\setminus } B_{\mathcal {M}}(p,\pi \mathrm {rch}(\mathcal {M}))$ is blue (color figure online)

We first note that if $|p-q| < 2 \mathrm {rch}(\mathcal {M})$, then *p* and *q* lie on the same connected component of $\mathcal {M}$. In fact we shall prove that if *p* and *q* lie on different connected components then $|p-q| \ge 2 \mathrm {rch}(\mathcal {M})$. Let $\mathcal {M}_1$ and $\mathcal {M}_2$ be two connected components of $\mathcal {M}$ with the smallest distance between them, if there is more than one such pair we pick one. We may assume that *p* lies on $\mathcal {M}_1$ and *q* on $\mathcal {M}_2$. Consider points the $x \in \mathcal {M}_1$ and $y \in \mathcal {M}_2$, where the distance $d(\mathcal {M}_1,\mathcal {M}_2)$ is attained (Fig. 14). The line segment [*xy*] is normal to both $\mathcal {M}_1$ and $\mathcal {M}_2$, from which we can conclude that the midpoint of [*xy*] is equidistant to both $\mathcal {M}_1$ and $\mathcal {M}_2$. Moreover there cannot be another connected component of $\mathcal {M}$ that is closer to the midpoint because we assumed that $\mathcal {M}_1$ and $\mathcal {M}_2$ are the two connected components that are the closest. This means that the midpoint lies on the medial axis. Our claim now follows. We can now safely assume that $\mathcal {M}$ has one connected component.

Thanks to Lemma 12, we know that if $d_\mathcal {M}(p,q) = \pi \mathrm {rch}(\mathcal {M})$, then $|p-q|\ge 2 \mathrm {rch}(\mathcal {M})$. This means that we can subdivide $\mathcal {M}$ in $B_{\mathcal {M}}(p,\pi \mathrm {rch}(\mathcal {M}))$, the geodesic ball of radius $\pi \mathrm {rch}(\mathcal {M})$, and $\mathcal {M}{\setminus } B_{\mathcal {M}}(p,\pi \mathrm {rch}(\mathcal {M}))$. Now suppose that $(\mathcal {M}{\setminus } B_{\mathcal {M}}(p,\pi \mathrm {rch}(\mathcal {M}))) \cap B(p, 2 \mathrm {rch}(\mathcal {M})) \ne \emptyset$, with $B(p, 2 \mathrm {rch}(\mathcal {M}))$ the open Euclidean ball of radius $2 \mathrm {rch}(\mathcal {M})$. We pick a point $y \in (\mathcal {M}{\setminus } B_{\mathcal {M}}(p,\pi \mathrm {rch}(\mathcal {M}))) \cap B(p, 2 \mathrm {rch}(\mathcal {M})$ that is the closest to *p*. We now see that

- [*yp*] is normal to $\mathcal {M}$ at *y* and thus for all $x \in [yp]$ with $|x-y| < \mathrm {rch}(\mathcal {M})$, $\pi _\mathcal {M}(x)= y$, by Federer’s tubular neighbourhood theorem.
- $$|y-p| < 2 \mathrm {rch}(\mathcal {M})$$

For any $0<\epsilon < \mathrm {rch}(\mathcal {M}) -|y-p|/2$, we can pick the point $x \in [yp]$ with a distance $\mathrm {rch}(\mathcal {M})-\epsilon$ from *y*. Due to Federer’s tubular neighbourhood theorem $\pi _\mathcal {M}(x) =y$ but by construction *x* closer to *p* than to *y*, a contradiction. It follows that $(\mathcal {M}{\setminus } B_{\mathcal {M}}(p,\pi \mathrm {rch}(\mathcal {M}))) \cap B(p, 2 \mathrm {rch}(\mathcal {M})) = \emptyset$. $\square$

Lemmas 13 and 14 immediately give us the following:

#### Corollary 6

**Fig. 15:**
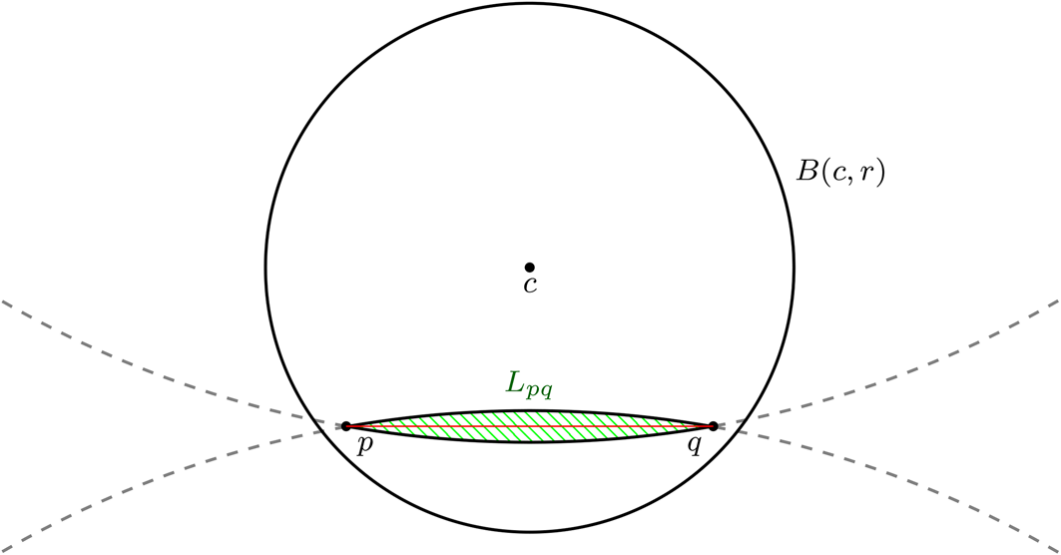
The lens shaped region $L_{pq}$ is indicated in green, the grey dashed circles have radius $\mathrm {rch}(\mathcal {M})$. We see that $L_{pq}\subset B(c,r)$ (color figure online)

A minimizing geodesic connecting *p* and *q* is contained in the lens shaped region $L_{pq}$, where $L_{pq}$ is constructed as follows. We first take the circle of radius equal to the reach $\mathrm {rch}(\mathcal {M})$, such that the line [*pq*] is a chord. This chord divides the circle in two parts. $L_{pq}$ is the hypersurface of revolution found by revolving the shortest part of the circle, denoted by $C_{s,[pq]}$, around [*pq*]. Alternatively $L_{pq}$ is also the intersection of all balls of radius reach such that [*pq*] is a cord on the boundary sphere of the ball (Fig. 15). $L_{pq}$ is also referred to as a spindle.

Let *B*(*c*, *r*) be a ball of radius $r < \mathrm {rch}(\mathcal {M})$ and let *p* and *q* now be any points in *B*(*c*, *r*). Eventually we shall again impose that *p* and *q* lie on $\mathcal {M}$, but we ignore this for the moment. We claim that $L_{pq}$ is completely contained in *B*(*c*, *r*). Consider any affine plane *P* spanned by containing [*pq*]. We look at the two circles of radius $\mathrm {rch}(\mathcal {M})$ in this plane, such [*pq*] is a chord. Because these circles of radius $\mathrm {rch}(\mathcal {M})$ have larger radius than the circle $B(c,r)\cap P$, the shortest parts of the circles of radius $\mathrm {rch}(\mathcal {M})$, namely $C_{s,[pq]}$ and its mirror image, lie inside $B(c,r)\cap P$.

We are now ready to prove the following theorem in the $C^2$ setting. The proof in the $C^{1,1}$-setting is given in Corollary 1.

#### Theorem 4

Let $\mathcal {M}$ be a compact $C^2$ manifold embedded in $\mathbb {R}^d$ and *B*(*c*, *r*) a ball of radius $r<\mathrm {rch}(\mathcal {M})$. Then $\mathcal {M}\cap B(c,r)$ is geodesically convex, in the sense that a minimizing geodesic connecting any two points in $\mathcal {M}\cap B(c,r)$ is itself contained in $\mathcal {M}\cap B(c,r)$.

#### Proof

For any two points $p,q \in \mathcal {M}\cap B(c,r)$, we consider the geodesic $\gamma (t)$ connecting *p* and *q*. As we have seen above $\gamma \subset L_{pq}$ and trivially $\gamma \subset \mathcal {M}$, so

$$\begin{aligned} \gamma \subset L_{pq} \cap \mathcal {M}\subset B(c,r) \cap \mathcal {M}. \end{aligned}$$

$$\square $$

## Conclusions and future research

Our characterization of the reach in terms of metric distortion does hold for arbitrary subsets of Euclidean space and is not restricted to the $C^2$ setting. For the bounds on variation of tangent spaces of manifolds with positive reach there is still a gap between the $C^{1,1}$ and smooth setting. Closing this gap is quite important as guarantees of many algorithms are based on these results.

## Footnotes

1 If there is more than one direction we simply pick one.

2 We can assume positivity without loss of generality, and, again, if there is more than one direction, we pick one.
